# Development of metabolic and inflammatory mediator biomarker phenotyping for early diagnosis and triage of pediatric sepsis

**DOI:** 10.1186/s13054-015-1026-2

**Published:** 2015-09-09

**Authors:** Beata Mickiewicz, Graham C. Thompson, Jaime Blackwood, Craig N. Jenne, Brent W. Winston, Hans J. Vogel, Ari R. Joffe, for the Alberta Sepsis Network

**Affiliations:** Bio-NMR Center, Department of Biological Sciences, University of Calgary, Calgary, AB Canada; Division of Emergency Medicine, Department of Pediatrics, University of Calgary, Calgary, AB Canada; Division of Pediatric Critical Care Medicine, Department of Pediatrics, University of Alberta, 4-546 Edmonton Clinic Health Academy; 11405 87 Avenue, Edmonton, AB T6G 1C9 Canada; Calvin, Phoebe and Joan Snyder Institute for Chronic Diseases, University of Calgary, Calgary, AB Canada; Department of Critical Care Medicine, University of Calgary, Calgary, AB Canada; Department of Medicine, University of Calgary, Calgary, AB Canada; Department of Biochemistry and Molecular Biology, University of Calgary, Calgary, AB Canada

## Abstract

**Introduction:**

The first steps in goal-directed therapy for sepsis are early diagnosis followed by appropriate triage. These steps are usually left to the physician’s judgment, as there is no accepted biomarker available. We aimed to determine biomarker phenotypes that differentiate children with sepsis who require intensive care from those who do not.

**Methods:**

We conducted a prospective, observational nested cohort study at two pediatric intensive care units (PICUs) and one pediatric emergency department (ED). Children ages 2–17 years presenting to the PICU or ED with sepsis or presenting for procedural sedation to the ED were enrolled. We used the judgment of regional pediatric ED and PICU attending physicians as the standard to determine triage location (PICU or ED). We performed metabolic and inflammatory protein mediator profiling with serum and plasma samples, respectively, collected upon presentation, followed by multivariate statistical analysis.

**Results:**

Ninety-four PICU sepsis, 81 ED sepsis, and 63 ED control patients were included. Metabolomic profiling revealed clear separation of groups, differentiating PICU sepsis from ED sepsis with accuracy of 0.89, area under the receiver operating characteristic curve (AUROC) of 0.96 (standard deviation [SD] 0.01), and predictive ability (*Q*^2^) of 0.60. Protein mediator profiling also showed clear separation of the groups, differentiating PICU sepsis from ED sepsis with accuracy of 0.78 and AUROC of 0.88 (SD 0.03). Combining metabolomic and protein mediator profiling improved the model (*Q*^2^ =0.62), differentiating PICU sepsis from ED sepsis with accuracy of 0.87 and AUROC of 0.95 (SD 0.01). Separation of PICU sepsis or ED sepsis from ED controls was even more accurate. Prespecified age subgroups (2–5 years old and 6–17 years old) improved model accuracy minimally. Seventeen metabolites or protein mediators accounted for separation of PICU sepsis and ED sepsis with 95 % confidence.

**Conclusions:**

In children ages 2–17 years, combining metabolomic and inflammatory protein mediator profiling early after presentation may differentiate children with sepsis requiring care in a PICU from children with or without sepsis safely cared for outside a PICU. This may aid in making triage decisions, particularly in an ED without pediatric expertise. This finding requires validation in an independent cohort.

**Electronic supplementary material:**

The online version of this article (doi:10.1186/s13054-015-1026-2) contains supplementary material, which is available to authorized users.

## Introduction

Sepsis is a leading cause of mortality in children worldwide [1]. In children age ≥1 year in the United States, sepsis is the second most common cause of death [2, 3]. The incidence of sepsis in children is increasing. Researchers in one study found an 81 % increase in hospitalizations for severe sepsis between 1995 and 2005 [4–6]. In addition, sepsis may lead to significant physical, neuropsychological, and neurocognitive morbidity in survivors [7–10]. For example, in a recent multicenter study, investigators found that 34 % of survivors of severe sepsis had a decline in their functional status at 28 days, and 18 % were determined to have a “poor” functional outcome (moderate, severe, or vegetative disability) [10].

Early, appropriate antibiotic therapy, fluid resuscitation, and vasoactive support are associated with improved outcomes after sepsis in a highly time-dependent manner [11–13]. The first steps in this therapy are the early diagnosis of sepsis followed by the appropriate stratification of patients (e.g., admission to a hospital ward for observation or to a more intensive monitoring environment in a critical care unit). These first steps are left to the physician’s judgment, as there is currently no accepted, accurate biomarker available to help in making this decision [13–15]. Physicians’ judgment in pediatric emergency departments (EDs) with experienced physicians and in tertiary pediatric intensive care units (PICUs) is likely quite different from that in smaller centers without extensive experience with sepsis in children [16]. The vast majority (>80 %) of children requiring emergency care present to an ED that does not have this specialized pediatric expertise [16, 17]. This problem has led to “great interest in developing diagnostic and stratification biomarkers for sepsis” [18]. A systems biology approach to biomarker phenotyping of the systemic response to sepsis has the potential to provide diagnostic and patient stratification profiles that inform clinical decision-making [19]. Unlike more proximal genomic and transcriptomic analysis, untargeted metabolomics and targeted proteomics reflect the downstream systems-level metabolic processes and pathways at play, defining specific phenotypes and shedding light on underlying pathophysiology [20, 21].

In previous work, our group has found that metabolomic modeling in adults accurately differentiated 39 patients with septic shock patients from 20 intensive care unit (ICU) control patients and that in children, it differentiated 58 PICU patients with septic shock from 39 PICU control patients [22, 23]. In adults, combining metabolomic and protein mediator data more accurately differentiated patients with septic shock from ICU controls [24]. In these studies, our group demonstrated promising metabolic biomarker profiles for diagnosing septic shock in ICU patients. In the present study, we asked different questions:

1. Is there a biomarker-defined phenotype that can differentiate children with sepsis who require intensive care from those who do not?

2. Does combining metabolic profiling with an analysis of protein mediators improve modeling?

3. Are there a limited number of biomarkers that may be used for targeted phenotyping in future studies?

As there is currently no objective gold standard for making this decision in the clinic, we used the judgment of experienced regional pediatric ED and PICU attending physicians as the standard for triage location of care. This standard is the same as that used for telemedicine and pediatric transport systems, where the expertise at the specialized center is relied upon [16].

## Methods

### Ethical approval

This study was approved by the Health Research Ethics Board of the University of Alberta (Pro00008797) and the Conjoint Health Research Ethics Board of the University of Calgary (Ethics ID 23426).

### Patient cohorts

#### PICU sepsis cohort

The Alberta Sepsis Network (ASN) prospectively enrolled all eligible children up to age 17 years admitted to the only two PICUs in the Province of Alberta, Canada, with a diagnosis of sepsis between April 2010 and October 2013. *Sepsis* was defined as the systemic inflammatory response syndrome (SIRS) caused by a suspected or proven bacterial or fungal infection [25], with antibiotics ordered and an arterial and/or central venous line in place. Patients not expected to survive ≥24 h, refusing intubation or vasoactive infusions (i.e., palliative care), or already having had severe sepsis for ≥48 h (defined as sepsis with cardiovascular dysfunction, acute respiratory distress syndrome, or two other organ dysfunctions) [25] were excluded. Demographic, infection and severity-of-illness variables (including pediatric logistic organ dysfunction [PELOD] and Pediatric Risk of Mortality [PRISM III] scores) were recorded prospectively [26, 27]. Blood was drawn as soon as possible on the day the patient met the eligibility criteria, using deferred consent. If consent was subsequently refused, the blood was not used and the patient was not enrolled. Patients were divided into the predefined 2–5-year-old and 6–17-year-old age groups on the basis of suspected pathophysiology, as done by others [23, 25], with microbiologically confirmed sepsis (positive culture from a normally sterile site, including blood, cerebrospinal fluid, peritoneal fluid, or tissue) or pneumonia without microbiological confirmation (SIRS with chest infiltrate suggestive of pneumonia). We did not consider sputum or endotracheal aspirates as sterile site cultures or require them as confirmatory of pneumonia, and it is not current practice to perform bronchoalveolar lavage in patients with suspected pneumonia. The site of infection was defined as that diagnosed by the attending medical team.

#### ED sepsis cohort

The ASN prospectively enrolled all children age ≤17 years admitted to one pediatric ED in Alberta with a diagnosis of sepsis. *Sepsis* was defined as SIRS caused by a suspected or proven bacterial or fungal infection [25], with antibiotics and blood culture ordered. Patients who were admitted to the PICU from the ED were included in the PICU sepsis cohort and not in the ED sepsis cohort. Basic demographic information was recorded, and blood was drawn as soon as possible on the day the patient met the eligibility criteria after informed consent was obtained. The site of infection was defined as that diagnosed by the attending medical team.

#### ED control cohort

In the same pediatric ED, all previously healthy children age ≤17 years admitted for a procedure and without an infection (i.e., no history of fever within 2 weeks and no clinical evidence of infection) were prospectively enrolled. These children had traumatic lacerations, fractures and/or dislocations, or foreign body removal requiring intravenous sedation and/or analgesia. Blood was drawn after consent was obtained, and basic demographic information was recorded.

### Sample collection and preparation

Samples were obtained via an existing arterial or central venous catheter (PICU sepsis cohort) or an intravenous tube insertion or blood culture draw (ED cohorts). Sample processing was performed as described in Additional file 1.

### Proton nuclear magnetic resonance spectroscopy and metabolite concentration profiling

Proton nuclear magnetic resonance (^1^H-NMR) spectra were acquired on a Bruker AVANCE-II 600 MHz spectrometer (Bruker BioSpin, Milton, ON, Canada). All spectra were randomly ordered for untargeted profiling to avoid progressive bias. Untargeted profiling involves identification of different compounds by their characteristic spectral signature using information that is stored in an external metabolite reference database [28–30]. Detailed methods are described in Additional file 1.

### Protein mediator profiling

Quantification of targeted protein mediators (cytokines, chemokines, and acute-phase proteins involved in inflammation) was done using validated Luminex bead-based multiplexing assays according to manufacturer’s instructions (Luminex, Austin, TX, USA). Detailed methods are described in Additional file 1.

### Statistical modeling

Multivariate statistical analysis was performed using the SIMCA-P+ software (v12.0.1; Umetrics, Umeå, Sweden) [19, 31–35]. All metabolites or protein mediators with >50 % missing values were excluded from analysis. Data preprocessing (median fold change normalization, logarithmic transformation, centering, and unit variance scaling) was first conducted separately for the metabolic and protein mediator datasets and then for the combined dataset [33]. An unsupervised principal component analysis (PCA) was performed to obtain an overview of the multivariate dataset and to identify and exclude outliers that could seriously disturb the supervised models [31]. Outliers are defined as those samples that are situated outside the 95 % confidence interval of the Hotelling’s *T*^2^ distribution (elliptical or spherical area in the score scatterplot) [31]. Then, supervised partial least squares discriminant analysis (PLS-DA) and orthogonal PLS-DA (OPLS-DA) models were developed to determine the best class discrimination (PICU sepsis, ED sepsis, and ED control) based on the preprocessed original data [31–35]. The OPLS-DA method was applied to models including only two classes. The OPLS-DA models for the combined datasets were based on potentially relevant metabolites and protein mediator data selected using the variable importance to projection (VIP), and only those variables with VIP >1 were chosen [31]. In supervised analysis, *R*^2^Y (the percentage of variation explained by the model) and *Q*^2^ (the predictive ability of the model) metrics were calculated using a sevenfold cross-validation method [31, 36]. Additionally, the OPLS-DA models were validated by calculating coefficient of variation-analysis of variance *p* values and the receiver operating characteristic curve (ROC) (Metz ROC Software; University of Chicago, Chicago, IL USA) [37, 38]. The sensitivity, specificity, and accuracy were determined on the basis of sample class prediction during sevenfold cross-validation (Y-predcv) using SIMCA-P+ software. To describe specific biopatterns for 2–17-year-old children using combined NMR and protein mediator data, the OPLS-DA regression coefficients were calculated and metabolites and/or protein mediators with significant changes in concentration (*p* <0.05) were considered as the most important variables [31].

## Results

### Description of the cohorts

The demographics, sites of infection, and severity-of-illness measures for each age and category cohort are given in Table 1. Children meeting ASN eligibility were prospectively entered into the PICU-ASN database after deferred consent was obtained (refusal rate of 22 %). Of 205 patients in the PICU-ASN database, those under 24 months of age (*n* =63), with missing data (*n* =3), or without clear bacterial sepsis (unknown, viral or other causes of SIRS) were excluded from this PICU sepsis nested cohort. The PICU sepsis cohort patients had PELOD and PRISM III severity-of-illness scores comparable to other PICU sepsis trial patients [39, 40]. Most PICU patients were ventilated (for about 1 week) and received supportive vasoactive infusions on the first day of sepsis. A significant number required renal replacement therapy and extracorporeal life support during their PICU stay. The average PICU length of stay after sepsis was about 1 week. The ED sepsis cohorts showed comparable demographics, except for having fewer comorbidities and better values for platelet count and systolic blood pressure. The ED control cohorts had proportionately more male subjects. Only 3 (3 %) of 94 of the PICU sepsis cohort were admitted to the PICU from the ward; these 3 patients had blood for analysis drawn in the ED, and none were outliers in the models.

**Table 1:** Description of the three cohorts of patients

Descriptive variable	PICU sepsis cohort		ED sepsis cohort		ED control cohort	
	2–5 yr	6–17 yr	2–5 yr	6–17 yr	2–5 yr	6–17 yr
	(*n* =36)	(*n* =58)	(*n* =43)	(*n* =38)	(*n* =25)	(*n* =38)
Age (mo)	39 (13.8)	138 (45)	37 (12.9)	131 (47)	45 (13)	133 (46)
Males	18 (50 %)	34 (59 %)	20 (47 %)	18 (47 %)	16 (64 %)	28 (74 %)
Weight (kg)	14.4 (4.5)	40.2 (20.5)	15.6 (3.4)	41.3 (22.5)	17.2 (3.0)	45.7 (17.6)
Underlying comorbidity					N/A	N/A
Neuromuscular	10 (28 %)	21 (36 %)	3 (7 %)	4 (11 %)		
Cardiac	9 (25 %)	7 (12 %)	0	1 (3 %)		
Respiratory	5 (14 %)	12 (21 %)	2 (5 %)	0		
PRISM III score	11 (9); 10 [2–18]	8 (7); 7 [3–11]	N/A	N/A	N/A	N/A
PELOD score	16.7 (9.4); 13 [11–22]	11.5 (7.6); 12 [10–13]	N/A	N/A	N/A	N/A
WBC count (10^9^/Litre)	12.9 (10.3)	13.6 (8,7)	12.8 (7.9); *n* =39	13.7 (7.2)	N/A	N/A
Platelet count (10^9^/Litre)	185 (124)	191 (127)	277 (99); *n* =39	252 (93)	N/A	N/A
Creatinine (μmol/L)	53 (48)	60 (39)	28 (7); *n* =30	47 (19); *n* =30	N/A	N/A
Lactate (mmol/L)	2.1 (1.9)	2.4 (2.5)	1.3 [1.2–1.6]; *n* =13	1.2 [1.0–2.0]; *n* =8	N/A	N/A
Lowest SBP (mmHg)	77 (13)	92 (17)	96 (13)	106 (11)	104 (14)	121 (12)
Lowest MAP (mmHg)	54 (8)	63 (13)	–	–	–	–
pH	7.3 (0.1)	7.3 (0.1)	–	–	–	–
Sepsis developed after first PICU day	6 (17 %)	4 (7 %)	N/A	N/A	N/A	N/A
Site of infection					N/A	N/A
Pneumonia without microbiological confirmation	21 (58 %)	28 (48 %)	23 (53 %)	11 (29 %)	–	–
Microbiologically confirmed (culture positive)	15 (42 %)^a^	30 (52 %)^b^	12 (28 %)^c^	19 (50 %)^d^	–	–
Clinically diagnosed	0 (0 %)	0 (0 %)	8 (19 %)^c^	8 (21 %)^d^	–	–
Mechanical ventilation on first day	27 (75 %)	38 (67 %)	N/A	N/A	N/A	N/A
Inotrope/vasopressor infusion on first day	20 (56 %)	34 (57 %)	N/A	N/A	N/A	N/A
Duration of mechanical ventilation after enrollment (days)	*n* =28	*n* =42	N/A	N/A	N/A	N/A
	(78 %)	(69 %)				
	10 [5–13]	6 [3–8]				
PICU length of stay after enrollment (days)	<2 days: 2 (6 %)	<2 days: 4 (7 %)	Hospital stay	Hospital stay	N/A	N/A
	8 [3–15]	7 [3–10]	4 [3, 5]; *n* =20	4 [3, 6]; *n* =19		
RRT	4 (11 %)	2 (4 %)	0	0	N/A	N/A
ECLS therapy	6 (17 %)	4 (7 %)	0	0	N/A	N/A
PICU mortality	1 (3 %)	1 (2 %)	0	0	0	0

*Abbreviations*: *ECLS* extracorporeal life support, *MAP* mean arterial pressure, *N*/*A* not applicable, *PELOD* pediatric logistic organ dysfunction, *PICU* pediatric intensive care unit, *PRISM* Pediatric Risk of Mortality, *RRT* renal replacement therapy, *SBP* systolic blood pressure; *WBC* white blood cells

Results are given as *n* (%) or mean (SD) or median [IQR].

^a^Sites of infection included meningitis (*n* =2), bacteremia (*n* =7), empyema (*n* =4), mediastinitis (*n* =1), and peritonitis (*n* =3)

^b^Sites of infection included meningitis (*n* =9), bacteremia (*n* =12), empyema (*n* =3), peritonitis (*n* =7), and fasciitis (*n* =1)

^c^Sites of infection included microbiologically confirmed meningitis (*n* =3), bacteremia (*n* =1), peritonitis (*n* =2), urinary tract infection (*n* =4), and *Streptococcus pyogenes* throat culture (*n* =2) and clinically diagnosed otitis media with draining ear or mastoiditis (*n* =3), cellulitis (*n* =2), cervical adenitis (*n* =1), and other (*n* =2)

^d^Sites of infection included microbiologically confirmed bacteremia (*n* =3), peritonitis (*n* =7), urinary tract infection (*n* =4), otitis media with *S. pyogenes* throat infection (*n* =5), clinically diagnosed otitis media with mastoiditis (*n* =2), osteomyelitis (*n* =1), pelvic abscess (*n* =1), toxic shock syndrome (*n* =1), and other (3)

### 2–17-year-old children

Ninety-four PICU sepsis, 81 ED sepsis, and 63 ED control patients were included. A total of 58 metabolites and 48 protein mediators were recognized and quantified for each patient (Additional file 1: Table E1). Totals of 1.12 % and 2.01 % missing values were observed in the NMR and protein mediator datasets, and these were randomly distributed.

### Profiling

#### Metabolomic profiling

**Fig. 1:**
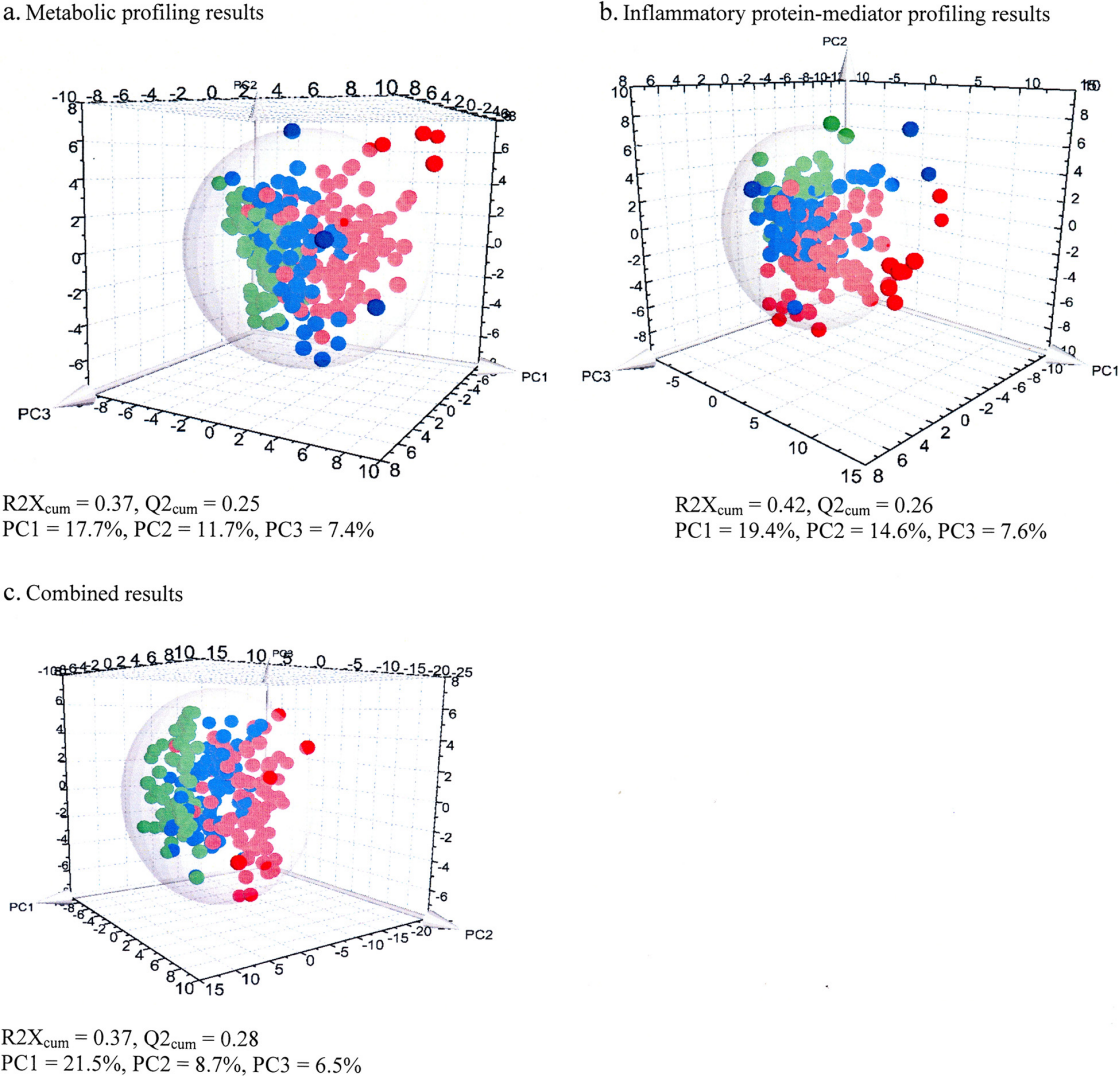
Principal component analysis (PCA) results for the 2–17-year-old cohorts based on the preprocessed original data (where each point represents one patient). **a** Metabolomic profiling data. **b** Inflammatory protein mediator profiling data. **c** Combined biomarker profiling data. The three-dimensional PCA score scatterplots show the distribution of observations (*red dots*, pediatric intensive care unit sepsis patients; *blue dots*, emergency department [ED] sepsis patients; *green dots*, ED controls) in the three-dimensional space formed by principal components PC1, PC2, and PC3. The PCs are the lines in the multivariable dimensional space (variables: metabolites, protein mediators) that best approximate the observations in the least squares sense. The sphere describes the 95 % confidence interval of the Hotelling’s *T* ^2^ distribution

**Fig. 2:**
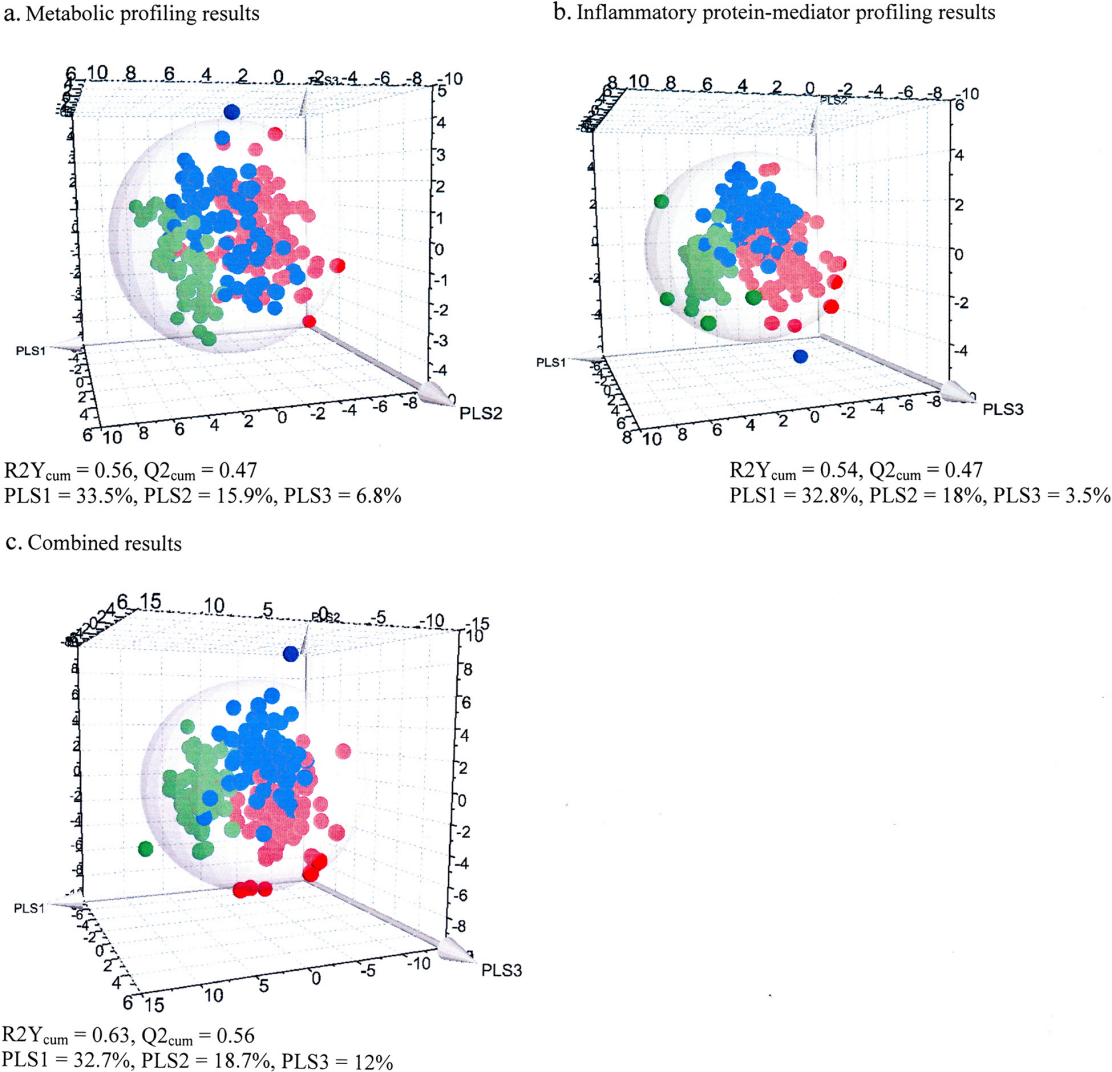
Partial least squares discriminant analysis (PLS-DA) for the 2–17-year-old cohorts based on the preprocessed original data. **a** Metabolomic profiling data. **b** Inflammatory protein mediator profiling data. **c** Combined biomarker profiling data. The three-dimensional PLS-DA score scatterplots show the distribution of observations (*red dots*, pediatric intensive care unit [PICU] sepsis patients; *blue dots*, emergency department [ED] sepsis patients; *green dots*, ED controls) in the three-dimensional space formed by PLS components (PLS1, PLS2, and PLS3). During the model construction, a discriminant plane (PLS component) was found in which the projected observations were well separated according to the class (PICU sepsis, ED sepsis, ED controls). The sphere describes the 95 % confidence interval of the Hotelling’s *T* ^2^ distribution

**Fig. 3:**
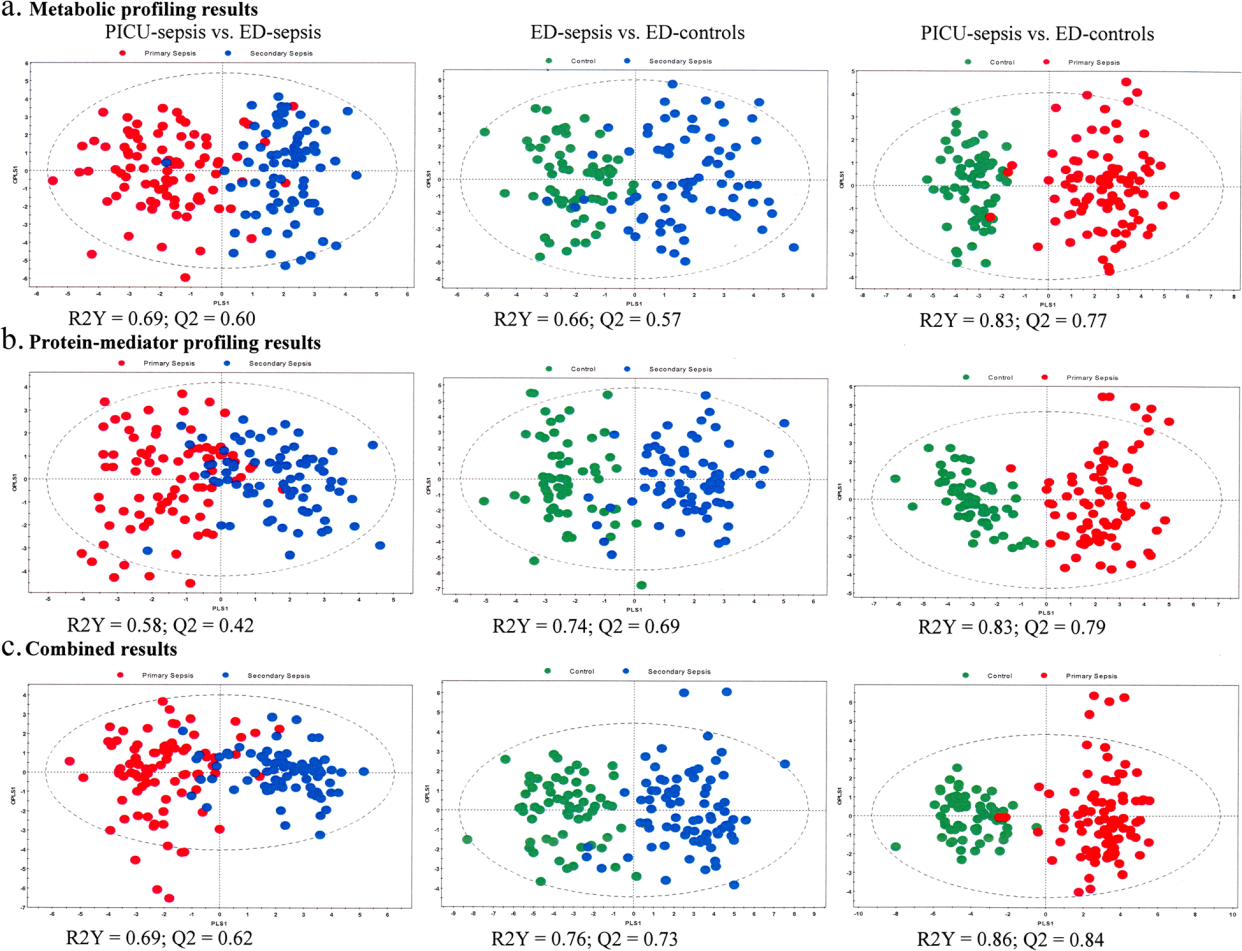
Orthogonal partial least squares discriminant analysis for the 2–17-year-old cohorts, using metabolomic profiling (**a**), protein-mediator profiling (**b**), and combined biomarker profiling (**c**) data. *Red dots*, pediatric intensive care unit (PICU) sepsis cohort (primary sepsis); *blue dots*, emergency department (ED) sepsis cohort (secondary sepsis); *green dots*, ED control cohort

Three principal components (PCs) were calculated via cross-validation to build the PCA model, with good data grouping, explaining the following percentages of variation: PC1 17.7 %, PC2 11.7 %, and PC3 7.4 % (Fig. 1a and Additional file 2: Figure E7a). There were nine outliers comprising 6 (6 %) in the PICU sepsis cohort and 3 (4 %) in the ED sepsis cohort. These outliers were excluded from subsequent analyses. A supervised PLS-DA showed that the three different cohorts were well clustered, with specific metabolic profiles for each. The model showed excellent goodness of fit (cumulative *R*^2^Y =0.56) and goodness of prediction (cumulative *Q*^2^ =0.47) (Fig. 2a). The OPLS-DA method was applied to compare metabolic variance in patient groups consisting of only two classes. The score scatterplots for each statistical analysis show clear separation of groups, with high values for the *R*^2^Y and *Q*^2^ parameters (Fig. 3a and Table 2). The predictive accuracy statistics for differentiating PICU sepsis from ED sepsis (Table 2) show accuracy of 0.89 and area under the ROC (AUROC) of 0.96 (standard deviation [SD] 0.01).

**Table 2:** Accuracy results of orthogonal partial least squares discriminant analysis models

Age group	Data	Outliers in PICU sepsis cohort, *n* (%)	*R* ^2^Y	*Q* ^2^	*p* Value	Sensitivity; specificity	PPV; NPV	Accuracy	AUROC (SD)
2–17 yr	Metabolites	6 (6 %)	0.69	0.60	3.9×10^−31^	0.86; 0.91	0.92; 0.86	0.89	0.96 (0.01)
	Mediators	13 (14 %)	0.58	0.42	4.9×10^−17^	0.79; 0.77	0.78; 0.78	0.78	0.88 (0.03)
	Combined	7 (7 %)	0.69	0.62	5.9×10^−33^	0.90; 0.85	0.87; 0.88	0.87	0.95 (0.01)
2–5 yr	Metabolites	3 (8 %)	0.68	0.50	4.4×10^−10^	0.76; 0.95	0.93; 0.83	0.87	0.95 (0.03)
	Mediators	6 (17 %)	0.67	0.45	1.8×10^−7^	0.73; 0.83	0.76; 0.81	0.79	0.91 (0.04)
	Combined	5 (14 %)	0.78	0.65	8.0×10^−15^	0.87; 0.93	0.90; 0.91	0.90	0.98 (0.02)
6–17 yr	Metabolites	6 (10 %)	0.79	0.68	2.0×10^−20^	0.94; 0.92	0.94; 0.92	0.93	0.97 (0.01)
	Mediators	9 (16 %)	0.67	0.45	4.5×10^−10^	0.86; 0.76	0.82; 0.80	0.81	0.91 (0.03)
	Combined	5 (9 %)	0.82	0.76	2.7×10^−25^	0.94; 0.92	0.94; 0.92	0.93	0.99 (0.01)

*Abbreviations*: *AUROC* area under the receiver operating characteristic curve, *NPV* negative predictive value, *PICU* pediatric intensive care unit, *PPV* positive predictive value, *SD* standard deviation

### Protein mediator profiling

PCA revealed three PCs explaining the following percentages of variation: PC1 19.4 %, PC2 14.6 %, and PC3 7.6 % (Fig. 1b and Additional file 2: Figure E7b). A total of 20 outliers comprising 13 (14 %) in the PICU sepsis cohort, 4 (5 %) in the ED sepsis cohort, and 3 (5 %) in the ED control cohort were excluded from further analyses. The PLS-DA model shows that the three different cohorts are reasonably well clustered, with an *R*^2^Y cumulative score of 0.54 and a *Q*^2^ cumulative score of 0.47 (Fig. 2b). The score scatterplots for each OPLS-DA statistical analysis show clear separation of groups, with high values for the *R*^2^Y and *Q*^2^ parameters (Fig. 3b and Table 2). The predictive accuracy statistics for differentiating PICU sepsis from ED sepsis (Table 2) showed accuracy of 0.78 and AUROC of 0.88 (SD 0.03), which were not as high as the values for NMR metabolomics.

#### Combined results

When PCA was performed, a three-PC model explained the following percentages of variation: PC1 21.5 %, PC2 8.7 %, and PC3 6.5 % (Fig. 1c and Additional file 2: Figure E7c). There were eight outliers comprising seven (7 %) in the PICU sepsis cohort and one (1 %) in the ED sepsis cohort. These outliers were excluded from subsequent analyses. A supervised PLS-DA shows that the three different cohorts are well clustered, with excellent model descriptive values: cumulative *R*^2^Y 0.63 and cumulative *Q*^2^ 0.56 (Fig. 2c). The score scatterplots for each OPLS-DA statistical analysis show clear separation of the groups, with high values for the *R*^2^Y and *Q*^2^ parameters (Fig. 3c and Table 2). The predictive accuracy statistics for differentiating PICU sepsis from ED sepsis (Table 2) show accuracy of 0.87 and AUROC 0.95 (SD 0.01). This model had very similar accuracy statistics compared with NMR alone; however, the sensitivity was higher (0.90 vs 0.86), the specificity was lower (0.85 vs 0.91), and goodness of prediction was higher (*Q*^2^ 0.62 vs 0.60).

### Models without outliers excluded

To confirm that the outliers detected in the PCA models (and subsequently excluded) did not bias the results of the supervised analyses, we recalculated the PLS-DA and OPLS-DA models including all outliers. This had only minor influence on the discriminative and predictive ability of the models (Additional file 1: Table E2). For example, for the combined dataset model differentiating PICU sepsis from ED sepsis, *R*^2^Y =0.67 and *Q*^2^ =0.63 compared with the model with outliers excluded where *R*^2^Y =0.69 and *Q*^2^ =0.62.

### Age subgroups

Full details of the results for the separate metabolomic and protein mediator analyses and the combined analyses in the 2–5-year-old and the 6–17-year-old children are given in Additional files 1 and 2. A summary of the results is shown in Table 2.

### Most meaningful metabolites and protein mediators

**Fig. 4:**
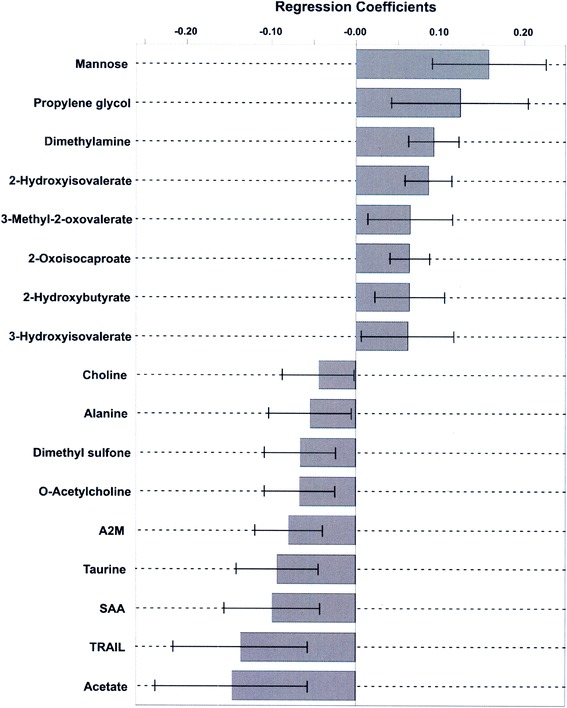
The regression coefficient plot for the orthogonal partial least squares discriminant analysis model differentiating pediatric intensive care unit (PICU) sepsis from emergency department (ED) sepsis cohorts in children ages 2–17 years (Fig. 3c). Positive values of the coefficients indicate increased concentrations in the PICU sepsis cohort samples, and negative values indicate a decrease in concentration in the PICU sepsis cohort samples, compared with the ED sepsis cohort samples. Only statistically significant metabolites and protein mediators are shown (*p* <0.05). *A2M* α-macroglobulin, *SAA* serum amyloid A, *TRAIL* tumor necrosis factor-related apoptosis-inducing ligand

Using the combined data, a total of 14 metabolites and 3 protein mediators defined the most significant differences responsible for the separation between PICU sepsis and ED sepsis cohorts in the OPLS-DA model (Fig. 4).

## Discussion

The main findings of this nested cohort comparison of three groups of patients ages 2–17 years with or without sepsis include the following. First, our metabolomic analysis using ^1^H-NMR spectroscopy clearly distinguished patients with sepsis requiring care in a PICU (*n* =94) from those with sepsis in the ED (*n* =81) and those without sepsis in the ED (controls, *n* =63) never requiring care in a PICU, with few outliers excluded from the model (6 % of the PICU sepsis cohort). Second, protein mediator profiling also distinguished between these groups; however, there were more outliers that had to be excluded from these analyses, limiting the accuracy of this method alone. Third, combining metabolomic and protein mediator data produced relatively few outliers (7 % of the PICU sepsis cohort), AUROC of 0.95 (SD 0.01), and the strongest model (*Q*^2^ =0.62), being more informative than either dataset alone. Fourth, the prespecified age subgroups resulted in only marginally more accurate models, but at the price of smaller patient numbers, and more outliers were excluded from model development. Fifth, a small group of 17 metabolites and protein mediators accounted for the separation of PICU sepsis and ED sepsis cohorts with 95 % confidence. Taken together, our data suggest that development of a laboratory test using these findings to help make diagnostic and triage decisions in children with sepsis is feasible.

These results are important for several reasons. First, they demonstrate proof of concept for using biomarker phenotyping in a clinical environment, generating information on patient biology that can guide clinicians’ diagnosis and triage decisions [19]. Second, the potential of this biomarker phenotyping has been demonstrated in a disease where decisions are time-sensitive [11–13]. In sepsis, poor decisions can mean the difference between survival and death and between survival with or without significant functional and/or neurocognitive sequelae [7–13]. For example, in meningococcemia, the symptoms in the first 4–6 h are non-specific, and many children are initially misdiagnosed by their physicians [41]. Third, these results were obtained in children, and regionalization of clinical experience in pediatric EDs and PICUs leaves most hospitals where children present without the specialized expertise to make timely diagnostic and triage decisions [16, 17]. Biomarker phenotyping accurately tracked the clinical decisions made by the specialized pediatric physicians, differentiating a PICU cohort that had high need for ventilation and vasoactive infusions and a prolonged PICU length of stay from an ED cohort that did not require the specialized and costly care of a PICU. Fourth, by using this discovery and systems biology–based approach, we identified a limited number of metabolites and protein mediators of interest that may realistically lead to development of a point-of-care decision aid and inform future research into the mechanisms of severe sepsis in children.

Whether the 17 metabolites and mediators of interest cause or are the result of manifestations of sepsis cannot be determined on the basis of the observational design of our study. Nevertheless, overall, they suggest broad changes in metabolic and inflammatory processes induced by severe sepsis, identified together as a specific biopattern to inform patient triage and possible pathophysiological mechanisms (see Additional file 1). For example, the metabolite changes suggest PICU sepsis–associated enhanced fatty acid breakdown, ketoacidosis, and dysfunction in amino acid metabolism [30]; hepatic glycogen catabolism [42]; and disruption in glycerophospholipid and sulfur metabolism [30]. The protein mediator changes suggest altered leukocyte recruitment and function [43–45]. A representative metabolic pathway network identified the most impaired biological pathways in PICU sepsis: taurine and hypotaurine metabolism; glycine, serine, and threonine metabolism; aminoacyl–transfer RNA biosynthesis; pyruvate metabolism; and, in 6–17-year-olds, arginine and proline metabolism [46]. This is similar to the most perturbed pathways in a previous PICU septic shock (vs PICU control) cohort [23].

This study has some limitations. It was performed at two centers, and the ED cohorts were from one center, possibly limiting the generalizability of the results. The number of patients included in each cohort was modest. The exact timing of the onset of sepsis in patients is unclear, as some present at different times in the course of the systemic response. In addition, the time from presentation until blood was drawn was likely different in the PICU sepsis and ED sepsis cohorts. We cannot be sure if different models would be obtained if stricter criteria for time course were applied. The PICU and ED sepsis cohorts were broadly separated by age groups a priori, but not strictly matched for age, sex, and clinical severity, and the definition of sepsis was more stringent in the PICU cohort. The ED control group was meant to reflect children with acute stress known not to be due to infection; thus, that group included children with predominantly fractures and lacerations. We did not determine whether the model could differentiate PICU sepsis and ED sepsis from other non-sepsis diagnoses that may require intensive care. Nevertheless, these patients were recruited from the only two PICUs serving the Province of Alberta and much of Northern Canada, with a catchment population >4 million. The biomarker phenotyping was applied in the real-world setting of patients presenting to the hospital with sepsis, regardless of the exact time of onset of their disease. Finally, the number of patients included is the largest sample for biomarker phenotyping in children of which we are aware [18, 19, 22, 23, 47].

We did not compare our biomarker phenotyping with existing Pediatric Early Warning Scores (PEWSs) used in the ED. We do not believe that this is a major limitation, for several reasons. First, most of these scores include, in addition to vital signs, subjective descriptions of the level of consciousness, capillary refilling, work of breathing, and worry about clinical status [48]. The goal of our model is to allow decisions that do not rely on this subjective expertise. Second, evaluations of the existing PEWSs have concluded that they are not accurate enough to replace clinical judgment, having inadequate discriminant ability for predicting PICU admission [48–51]. A related limitation is that we did not determine whether PICU sepsis patients initially presented with obvious fluid-refractory or vasoactive-dependent sepsis making a biomarker unnecessary. We retrospectively determined timing of interventions in ASN patients admitted to one PICU and found that, in patients ventilated on day 1 of sepsis, the times from initial presentation to 20 ml/kg volume bolus or vasoactive infusions were, on average, >3 h and >8 h, respectively. This suggests that few patients were declared to have fluid-refractory or vasoactive-dependent septic shock in the first hours after presentation to the ED.

A prospective validation of our findings in an independent multicenter cohort using clear definitions of sepsis upon presentation to an ED is needed. Although there is need for some caution [52, 53], we believe that developing a point-of-care test targeted at detecting the metabolites and protein mediators of interest identified here holds great promise, as recently found for gene expression mosaics [54]. This may involve enzyme-linked immunosorbent assay or novel, rapid liquid chromatography-mass spectrometry techniques [55, 56].

## Conclusions

In children ages 2–17 years, combining metabolomic and inflammatory protein mediator profiling early after presentation can differentiate children with sepsis requiring care in a PICU from children with or without sepsis who can be safely cared for outside a PICU. By using this discovery and systems biology–based approach, we identified a limited number of metabolites and protein mediators of interest that may realistically lead to development of a point-of-care decision aid. This may aid triage decisions, particularly in EDs without pediatric expertise. This finding requires validation in an independent cohort.

## Key messages

• In children ages 2–17 years, combining metabolomic and inflammatory protein mediator profiling on serum and plasma early after presentation can differentiate children with sepsis requiring care in a PICU from children with or without sepsis who can be safely cared for outside a PICU.

• By using this discovery and systems biology–based approach, we identified a limited number of metabolites and protein mediators of interest that may realistically lead to development of a point-of-care decision aid.

## Additional files

Additional file 1:
**Supplemental descriptions of methods, statistical modeling, results of subgroups, discussion of the importance of the metabolites and protein mediators of interest (biomarkers) identified, and supplemental references.** This file also includes two tables. **Table E1.** The metabolites (non-targeted ^1^H NMR spectroscopy and metabolite concentration profiling) and protein mediators (targeted bead-based multiplex assay) detected and quantified. **Table E2.** Comparison of statistical measures calculated for the supervised OPLS-DA models without excluding outliers from the results presented in the main text of the article. (PDF 307 kb) Additional file 2:
**Results of the metabolomic and protein mediator biomarker phenotyping in the two age subgroups.** This file contains six figures depicting the results for the age subgroups and a seventh figure showing the loading plots that demonstrate which metabolites and/or inflammatory protein mediators contribute most to each component in the PCA models for the age 2–17-year-old cohort. (PDF 4020 kb)

## Abbreviations

ASN: Alberta Sepsis Network
AUROC: area under the receiver operating characteristic curve
ECLS: extracorporeal life support
ED: emergency department
^1^H-NMR: proton nuclear magnetic resonance
ICU: intensive care unit
MAP: mean arterial pressure
NPV: negative predictive value
OPLS-DA: orthogonal partial least squares discriminant analysis
PC: principal component
PCA: principal component analysis
PELOD: pediatric logistic organ dysfunction score
PEWS: Pediatric Early Warning Score
PICU: pediatric intensive care unit
PLS-DA: partial least squares discriminant analysis
PPV: positive predictive value
PRISM: Pediatric Risk of Mortality
ROC: receiver operating characteristic curve
RRT: renal replacement therapy
SBP: systolic blood pressure
SD: standard deviation
SIRS: systemic inflammatory response syndrome
VIP: variable importance to projection
WBC: white blood cells
