# Supplementary material for: Development of metabolic and inflammatory mediator biomarker phenotyping for early diagnosis and triage of pediatric sepsis
Source: Crit Care. 2015 Sep 9;19(1):320. doi: 10.1186/s13054-015-1026-2 (PMC4563828; doi:10.1186/s13054-015-1026-2)
Supplement: Additional file 1: — Supplemental descriptions of methods, statistical modeling, results of subgroups, discussion of the importance of the metabolites and protein mediators of interest (biomarkers) identified, and supplemental references. This file also includes two tables. Table E1. The metabolites (non-targeted 1H NMR spectroscopy and metabolite concentration profiling) and protein mediators (targeted bead-based multiplex assay) detected and quantified. Table E2. Comparison of statistical measures calculated for the supervised OPLS-DA models without excluding outliers from the results presented in the main text of the article. (PDF 307 kb) [file 13054_2015_1026_MOESM1_ESM.pdf]

Early diagnosis and triage of pediatric sepsis using metabolic and inflammatory mediator biomarkers.

Authors: Beata Mickiewicz PhD, Graham Thompson MD, Jaime Blackwood MD, Craig N Jenne PhD, Brent W Winston MD, Hans J Vogel PhD, Ari R Joffe MD, for the Alberta Sepsis Network.
Journal: Critical Care
Corresponding Author: Ari R Joffe MD; University of Alberta, Edmonton, AB, Canada; email: ari.joffe@albertahealthservices.ca

Additional File 1.

Supplemental descriptions of: Methods; Statistical Modeling; Results of Subgroups; Discussion of the importance of the metabolites-and-protein-mediators-of-interest (biomarkers) identified; and Supplemental References.

TableE1. The metabolites (non-targeted ^{1}H NMR Spectroscopy and Metabolite Concentration Profiling) and protein-mediators (targeted bead-based multiplex assay) detected and quantified.

TableE2. Comparison of statistical measures calculated for the supervised OPLS-DA models without excluding outliers to the results presented in the main manuscript.

I. Methods:

Sample Collection and Processing:

For metabolite analysis, 2ml (in age 2-14yr) to 4ml (in age over 14yr) of blood was collected into a serum tube, allowed to clot at room temperature for 60 minutes, and then placed on ice for processing within 2 hours of collection. The specimen was spun for 10 min at 2500 rpm in a refrigerated centrifuge, the serum transferred to a 4ml cryovial, and immediately frozen at -70°C. For inflammatory mediator profiling, 2ml (in age 2-14yr) to 4ml (in age over 14yr) of blood was collected into a sodium-heparin containing vacutainer tube. Immediately after collection, blood samples were gently inverted several times, and spun at 1,200 g for 15 min at 4°C in a swinging bucket centrifuge to separate plasma from the cellular fraction. Plasma was transferred to a 4ml cryovial, and immediately frozen at -70°C. Both plasma and serum samples underwent one extra freeze thaw cycle for aliquoting and distribution for batched analysis. Processing was performed by CCEPTR (Critical Care Epidemiologic and biologic Tissue Resource, a critical care tissue bank at the University of Calgary).

^{1}H NMR Spectroscopy and Untargeted Metabolite Concentration Profiling:

^{1}H NMR spectra were acquired on a Bruker AVANCE-II 600 MHz spectrometer (Bruker BioSpin Ltd., Milton, ON, Canada) using a standard presaturation pulse sequence (noesypr1d) with a mixing time of 100ms.[1,2] The ^{1}H NMR spectra were imported into Chenomx NMR Suite 7.5 software (Chenomx, Edmonton, AB, Canada) where phasing was performed manually followed by deletion of the water region and baseline correction. All spectra were randomly ordered for targeted profiling to avoid progressive bias.[1] Targeted profiling involves the identification and quantification of different compounds by their characteristic spectral signature using information that is stored in an external metabolite reference database. The concentration of 4,4-dimethyl-4-silapentane-1-sulfonic acid was used as a reference to determine the metabolite concentrations. All profiled metabolite NMR chemical shift assignments were confirmed using total correlation spectroscopy and ^{1}H,^{13}C heteronuclear single quantum coherence spectroscopy and then compared with the Human Metabolome Database (version 2.5).[3] If specific metabolite peaks could not be distinguished from the noise in the NMR spectra, the peaks were assigned

a zero value and considered as missing data. This untargeted approach to metabolomics identified 58 metabolites for analysis (Table E1).

Protein-Mediator Targeted Profiling:

Methods describing the analysis of protein-mediators by Luminex technology have been previously reported in detail.[4] All samples were run by an investigator blinded to cohort allocation to avoid potential bias. Quantification of protein-mediators (cytokines, chemokines, and acute phase proteins involved in inflammation) was done using validated Luminex bead-based multiplexing assays according to manufacturer's instructions. Targeted profiling was for 48 specific protein-mediators (Table E1). Assays consisted of the following arrays: Bio-Plex Pro™ Human Cytokine 21-plex Assay, Bio-Plex Pro™ Human Cytokine 27-plex Assay, and Bio-Plex Pro™ Human Acute Phase 5 + 4-plex Panel Complete (Bio-Rad Laboratories). Briefly, plasma samples were thawed quickly at 37°C, centrifuged at 20800g at 4°C for 10-minutes, and then stored on ice. Samples were diluted as recommended by the manufacturer and were distributed to appropriate wells of a 96 well flat bottom plate. The plate was then incubated, in the dark, for two-hours at room temperature on a plate shaker set at 500-rotations-per-minute (rpm). Wells were first washed three times with 100 µL of wash buffer and then 25 µL of diluted biotin antibody cocktail was added. The plate was subsequently incubated, in the dark, for one-hour at room temperature on a plate shaker set at 500-rpm. Wells were subsequently washed three times with 100 µL of wash buffer. Thereafter, 25 µL of diluted Streptavidin-PE was added to each well and the plate was incubated for 30-minutes at room temperature on a plate shaker set at 500-rpm. Wells were then washed three times with 100 µL of wash buffer. Subsequently, 75 µL of wash buffer was added to each well and the plate was read using a Luminex 200 apparatus (Applied Cytometry Systems, UK). The data were analyzed with StarStation V.2.3 (Applied Cytometry Systems),

II. Statistical Modeling:

Multivariate statistical analysis was performed using the SIMCA-P+ software (v12.0.1; Umeterics, Umeå, Sweden).[5-10] All metabolites or protein-mediators with more than 50% missing values (below the detection limit**,** the coefficient of variance between two replicates was more than 20% for protein-

mediators, or metabolite peaks that could not be distinguished from the noise in the NMR spectra) were excluded from analysis. Data pre-processing (median fold change normalization, logarithmic transformation, centering and unit variance scaling) was first conducted separately for the metabolic and protein-mediator datasets and then for the combined dataset.[8] An unsupervised Principal Component Analysis (PCA) was performed to obtain an overview of the multivariate dataset, summarize the major source of variation in the data, reveal possible grouping of observations, and to identify and exclude outliers (that could seriously disturb the supervised models).[6] Outliers are defined as those samples that are situated outside of the 95% confidence interval of the Hotelling's T-squared distribution (elliptic or spherical area in the score scatter plot).[6] In order to provide information about the metabolites and protein-mediators that contribute most to each component in the PCA models the PCA loadings plots were also constructed.

Then, supervised Partial Least Squares Discriminant Analysis (PLS-DA) and Orthogonal PLS-DA (OPLS-DA) models were developed to determine the best class discrimination (PICU-sepsis, ED-sepsis, ED-control) based on the preprocessed original data.[6,7,9] Briefly, both supervised methods are focused on data discrimination and classification. The objective of PLS-DA is to create a model that separates classes of observations on the basis of their x-variables (metabolites and protein-mediators). The OPLS-DA method is a modification of the PLS-DA model in which the systematic variation in the dataset is separated into two parts, one that is related to the class identification (and, therefore predictive) and the other that is unrelated (orthogonal). This kind of partitioning of the data improves model transparency and interpretability.[9,11] The OPLS-DA method was applied to models including only two classes. The OPLS-DA models for the combined datasets were based on potentially relevant metabolites and protein-mediator data selected using the variable importance to projection (VIP); only those variables with VIP>1 were chosen.[6] The VIP parameters summarize the importance of the x-variables (in our study the metabolites and inflammatory-protein-mediators) for the supervised models. The sum of squares of all VIP values is equal to the number of x-variables in the model, hence the average VIP is equal to 1. This means that if all x-variables have the same contribution to the model, they will have a VIP=1. The x-

variables with large VIP (VIP>1) are the most relevant for explaining the classes of observations, and generally VIP values below 0.5 are considered as irrelevant variables.[6,12]

To evaluate the PLS-DA and OPLS-DA models, R2Y (the percentage of variation explained by the model) and Q2 (the predictive ability of the model) metrics were calculated using a sevenfold cross-validation method.[9,11] In this case, 7 models were built with exactly one seventh of the data excluded from each model and each sample excluded a single time. The ability of the models to predict those samples not involved in the modeling provided a measure of the overall predictive ability of the model. This cross-validation method is a practical and reliable way to test the significance of the supervised models, and has become a standard in metabolomics analysis.[13-15] Additionally, the OPLS-DA models were validated by calculating CV-ANOVA p-values and the receiver operator characteristics (ROC) (Metz ROC Software, Chicago, IL USA).[16,17] The sensitivity, specificity, and accuracy were determined on the basis of sample class prediction during sevenfold cross-validation (Y-predcv) using the SIMCA-P+ software. During model construction the PICU-sepsis samples were assigned a response value of one, while the ED-sepsis samples were given a response of zero. If the predicted response value calculated during cross-validation was more than 0.5, the sample was assigned as a PICU-sepsis sample. Otherwise the sample was assigned as ED-sepsis. Based on this analysis the true positive, true negative, false positive and false negative cases were calculated. This is the recommended method for metabolomics data, and has been used by our group in adult sepsis models.[16-18] To describe specific biopatterns within the OPLS-DA model for 2-17y children using combined NMR and protein-mediator data, the OPLS-DA regression coefficients were calculated, and metabolites/protein-mediators with significant changes in concentration ($p<0.05$) were considered as the most important variables.[6]

III. Results of Subgroups:

2-5 year old children:

There were 36 PICU-sepsis, 43 ED-sepsis, and 25 ED-control patients. A total of 58 metabolites and 48 protein-mediators were recognized and quantified for each cohort.

Metabolic profiling: Three principal components were calculated via CV to build the model explaining the following percentage of variation: PC1 19.7%, PC2 14.5%, and PC3 8.4% (Figure E1a). There were 4 outliers, 3 (8%) from the PICU-sepsis cohort and 1 (2%) from the ED-sepsis cohort, and these were excluded from subsequent models. A supervised PLS-DA model shows the three different cohorts are well clustered, with specific metabolic profiles for each (R2Y cumulative 0.61, Q2 cumulative 0.43) (Figure E2a). An OPLS-DA method was applied to compare metabolic variance in patient groups consisting of only two classes. The score scatter plots for each statistical analysis (Figure E3a) show clear separation of groups, with high values of R2Y and Q2 parameters (Table 2). The predictive accuracy statistics for differentiating PICU-sepsis from ED-sepsis (Table 2) show an accuracy of 0.87 and AUROC of 0.95 (SD 0.03).

Protein-mediator profiling: Three principal components were calculated via CV to build the model explaining the following percentage of variation: PC1 19.9%, PC2 15.9%, and PC3 8.3% (Figure E1b). There were 7 outliers, 6 (17%) from the PICU-sepsis cohort, and 1 (2%) from the ED-sepsis cohort, and these were excluded from subsequent models. A supervised PLS-DA model shows the three different cohorts are reasonably well clustered, with specific protein-mediator profiles for each (R2Y cumulative 0.55, Q2 cumulative 0.36) (Figure E2b). The score scatter plots for each OPLS-DA model (Figure E3b) show clear separation of groups, with high values of R2Y and Q2 parameters. The predictive accuracy statistics for differentiating PICU-sepsis from ED-sepsis (Table 2) show an accuracy of 0.79 and AUROC of 0.91 (SD 0.04), not quite as high as for NMR metabolomics.

Combined Results: There were 36 PICU-sepsis, 43 ED-sepsis, and 25 ED-control patients. Three principal components were calculated for the combined data via cross-validation to build the model explaining the following percentage of variation: PC1 21.7%, PC2 9.7%, and PC3 6.9% (Figure E1c). There were 6 outliers, 5 (14%) from the PICU-sepsis cohort, and 1 (2%) from the ED-sepsis cohort. These outliers were excluded from subsequent analyses. A supervised PLS-DA analysis shows the three different cohorts are well clustered, with R2Y cumulative 0.69, and Q2 cumulative 0.57 (Figure E2c). The score scatter plots for each OPLS-DA statistical analysis (Figure E3c) show clear separation of groups, with high values of R2Y and Q2 parameters. The predictive accuracy statistics for differentiating PICU-

sepsis from ED-sepsis (Table 2) show accuracy 0.90 and AUROC 0.98 (0.02), better than for metabolic or protein-mediator profiling alone.

6-17 year old children:

There were 58 PICU-sepsis, 38 ED-sepsis, and 38 ED-control patients. A total of 58 metabolites and 48 protein-mediators were recognized and quantified for each cohort.

Metabolic profiling: Three principal components were calculated via CV to build the model explaining the following percentage of variation: PC1 18.5%, PC2 12.5%, and PC3 7.7% (Figure E4a). There were 6 outliers, all from the PICU-sepsis cohort (10%), and these were excluded from subsequent models. A supervised PLS-DA model shows the three different cohorts are well clustered, with specific metabolic profiles for each (R2Y cumulative 0.62, Q2 cumulative 0.42) (Figure E5a). The score scatter plots for each OPLS-DA model (Figure E6a) show clear separation of groups, with high values of R2Y and Q2 parameters (Table 2). The predictive accuracy statistics for differentiating PICU-sepsis from ED-sepsis (Table 2) show accuracy 0.93 and AUROC 0.97 (SD 0.01).

Protein-mediator profiling: Three principal components were calculated via CV to build the model explaining the following percentage of variation: PC1 19.9%, PC2 14%, and PC3 8.7% (Figure E4b). There were 11 outliers, 9 (16%) from the PICU-sepsis cohort, 1 (3%) from the ED-sepsis cohort, and 1 (3%) from the ED-control cohort, and these were excluded from subsequent models. A supervised PLS-DA model shows the three different cohorts are reasonably well clustered, with specific mediator profiles for each (R2Y cumulative 0.54, Q2 cumulative 0.45) (Figure E5b). The score scatter plots for each OPLS-DA model (Figure E6b) show clear separation of groups, with high values of R2Y and Q2 parameters. The predictive accuracy statistics for differentiating PICU-sepsis from ED-sepsis (Table 2) show an accuracy of 0.81 and AUROC of 0.91 (SD 0.03), not quite as high as for NMR-based metabolomics.

Combined Results: There were 58 PICU-sepsis, 38 ED-sepsis, and 38 ED-control patients. PCA analysis using three principal components were calculated for the combined data to build a well-clustered model explaining the following percentage of variation: PC1 23.9%, PC2 10.2%, and PC3 5.7% (Figure E4c).

There were 7 outliers, 5 (9%) from the PICU-sepsis cohort, 1 (3%) from the ED-sepsis cohort, and 1 (3%) from the ED-control cohort, and these were excluded from subsequent analyses. A supervised PLS-DA analysis shows the three different cohorts are well clustered, with R2Y cumulative 0.70 and Q2 cumulative 0.61 (Figure E5c). The score scatter plots for each OPLS-DA statistical analysis (Figure E6c) show clear separation of the groups, with high values for the R2Y and Q2 parameters. The predictive accuracy statistics for differentiating PICU-sepsis from ED-sepsis (Table 2) show an accuracy 0.93 and AUROC 0.99 (0.01), which is again better than that for metabolic or protein-mediator profiling alone.

IV. Discussion of the importance of the metabolites-and-protein-mediators-of-interest (biomarkers) identified:

Whether the 17 metabolites-and-mediators-of-interest cause, or are the result of manifestations of sepsis cannot be determined from the observational design. Nevertheless, overall they suggest broad changes in metabolic and inflammatory processes induced by severe sepsis, identified together as a specific biopattern to inform patient triage and possible pathophysiologic mechanisms.

Increased levels of 2-hydroxyisovalerate, 3-methyl-2-oxovalerate, 2-oxoisocaproate, 2-hydroxybutyrate, and 3-hydroxyisovalerate point to enhanced fatty acids breakdown, ketoacidosis, and dysfunction in amino acid metabolism in PICU-sepsis patients.[3] Hepatic glycogen catabolism might be responsible for a high concentration of mannose.[19] A drop in acetate and alanine levels suggest amino acid oxidation in PICU-sepsis patients. Moreover, decreased concentrations of choline, O-acetylcholine, taurine, and dimethyl-sulfone indicate disruption in glycerophospholipid and sulfur metabolism.[3] Finally, high levels of dimethylamine and propylene glycol point to kidney and liver dysfunction in PICU-sepsis patients when compared to the ED-sepsis cohort.[3,20] Overall, these findings suggest broad changes in metabolic processes induced by severe sepsis in children.

We observed decreased levels of Serum amyloid A (SAA) in PICU-sepsis patients compared with ED-sepsis patients. Serum amyloid A has been reported to correlate with C-reactive protein in human patients with septic shock.[21] As the kinetics of SAA levels in previous reports appears to be very rapid,

with an early spike followed by a progressive return to baseline, differences observed between the PICU-sepsis and ED-sepsis cohorts may be due to the time of presentation of patients in the two cohorts being at different points in the progression of their disease (i.e. PICU patients may not present until they are further along in the sepsis course). Alpha$_2$-macroglobulin (A2M) levels in our study were lower in the PICU-sepsis cohort when compared to the ED-sepsis group. A2M has been reported to correlate positively with survival in human sepsis.[22] It has been suggested that this molecule is protective, enhancing leukocyte recruitment and function, and bacterial killing (including phagocytosis, and reactive oxygen species generation).[22] Plasma levels of soluble tumor necrosis factor related apoptosis inducing ligand (TRAIL) have also been associated with patient outcomes, with lower levels associated with worse outcomes in human sepsis.[23] It has been suggested that TRAIL is required to limit leukocyte infiltration and inflammation, reducing tissue damage and preserving organ function.[23] Our findings are in agreement with previous studies, with the patients in the PICU-sepsis cohort presenting with lower levels of TRAIL than those in the ED-sepsis group.

Based on the most significant metabolites (Figure 4), a representative metabolic pathway network was constructed to identify the most impaired biological pathways ($p<0.05$ and pathway impact >0.1) during severe sepsis (PICU-Sepsis cohort). The analysis was performed in the MetaboAnalyst 2.5 software.[24] The most perturbed pathways (based on the metabolic profiling data only) were as follows:

1. Age 2-17 year old children- taurine and hypotaurine metabolism; glycine, serine, and threonine metabolism; aminoacyl-tRNA biosynthesis; and pyruvate metabolism.
2. Age 2-5 year old children: taurine and hypotaurine metabolism; glycine, serine, and threonine metabolism; aminoacyl-tRNA biosynthesis; pyruvate metabolism; and lysine degradation.
3. Age 6-17 year old children: taurine and hypotaurine metabolism; glycine, serine, and threonine metabolism; pyruvate metabolism; and arginine and proline metabolism.

We then compared these pathway analyses with the data obtained in our previous study using ^{1}H-NMR metabolic profiling to differentiate 58 pediatric septic-shock patients from 39 PICU-control patients.[18] We performed a pathway analysis on the data from that publication, and found the most perturbed pathways in the PICU septic shock cohort as follows:

1. Age 2-5 year old children: taurine and hypotaurine metabolism
2. Age 6-11 year old children: arginine and proline metabolism.

We believe that this strengthens the findings in the current study by showing overlap in the most perturbed metabolic pathways in the separate cohorts of PICU sepsis patients.

V. Supplemental References:

1. Weljie AM, Newton J, Mercier P, Carlson E, Slupsky CM: **Targeted profiling: quantitative analysis of 1H NMR metabolomics data.** *Anal Chem*, 2006;**78(13):**4430-4442.

2. Nicholson JK, Foxall PJ, Spraul M, Farrant RD, Lindon JC: **750 MHz 1H and 1H-13C NMR spectroscopy of human blood plasma.** *Anal Chem,* 1995;**67(5):**793-811.

3. Wishart DS, Knox C, Guo AC, Eisner R, Young N, Gautam B, Hau DD, Psychogios N, Dong E, Bouatra S, Mandal R, Sinelnikov I, Xia J, Jia L, Cruz JA, Lim E, Sobsey CA, Shrivastava S, Huang P, Liu P, Fang L, Peng J, Fradette R, Cheng D, Tzur D, Clements M, Lewis A, De Souza A, Zuniga A, Dawe M, Xiong Y, Clive D, Greiner R, Nazyrova A, Shaykhutdinov R, Li L, Vogel HJ, Forsythe I: **HMDB: a knowledgebase for the human metabolome.** *Nucleic Acids Res,* 2009;**37:**D603-10.

4. Roberts DJ, Jenne CN, Ball CG, Tiruta C, Leger C, Xiao Z, Faris PD, McBeth PB, Doig CJ, Skinner CR, Ruddell SG, Kubes P, Kirkpatrick AW: **Efficacy and safety of negative pressure peritoneal therapy for reducing the systemic inflammatory response after damage control laparotomy (the Intra-peritoneal Vacuum Trial): a study protocol for a randomized controlled trial.** *Trials*, 2013;**14:**141.

5. Nicholson JK, Holmes E, Kinross JM, Darzi AW, Takats Z, Lindon JC: **Metabolic phenotyping in clinical and surgical environments.** *Nature*, 2012;**491:**384-391.

6. Eriksson L, Johansson E, Kettaneh-Wold N, Trygg J, Wikstrom C, Wold S: **Multi- and megavariate data analysis part 1: Basic principles and applications.** Umeå, Sweden, Umetrics AB, 2006; pp 425.

7. Eriksson L, Johansson E, Kettaneh-Wold N, Trygg J, Wikstrom C, Wold S: **Multi- and megavariate data analysis part 2: Advanced applications and method extensions.** Umeå, Sweden, Umetrics AB, 2006; pp 307.

8. van den Berg RA, Hoefsloot HC, Westerhuis JA, Smilde AK, van der Werf MJ: **Centering, scaling, and transformations: improving the biological information content of metabolomics data.** *BMC Genomics*, 2006;**7:**142

9. Trygg J, Holmes E, Lundstedt T: **Chemometrics in Metabonomics**. *J Proteome Res*, 2007;**6:**469-479.

10. Madsen R, Lundstedt T, Trygg J: **Chemometrics in metabolomics: a review in human disease diagnosis.** *Anal Chim Acta*, 2010;**659(1-2):**23-33.

11. Picard RR, Cook DR: **Cross-validation of regression models.** *J Am Stat Assoc*, 1984;**79:**575-583.

12. Galindo-Prieto B., Eriksson L., Trygg J: **Variable influence on projection (VIP) for orthogonal projections to latent structures (OPLS).** *J. Chemometrics*, 2014;**28:**623-632.

13. Dessi A, Marincola FC, Pattummeli MG, Ciccarelli S, Corbu S, Ossicini C, Fanos V, Agostino R: **Investigation of the ^{1}H-NMR based urine metabolomic profiles of IUGR, LGA and AGA newborns on the first day of life.** *J Matern Fetal Neonatal Med*, 2014;**27(S2):**13-19

14. Bahety P, Tan YM, Hong Y, Zhang L, Chan EC, Ee PL: **Metabotyping of docosahexaenoic acid - treated Alzheimer's disease cell model.** *PLoS One* 2014;**9(2):**e90123

15. Liu SY, Zhang RL, Kang H, Fan ZJ, Du Z: **Human liver tissue metabolic profiling research on Hepatitis B virus-related hepatocellular carcinoma.** *World J Gastroenterol,* 2013;**19(22):**3423-3432.

16. Fawcett T: **An introduction to ROC analysis**. *Pattern Recognit Lett,* 2006;**27:**861-874.

17. Zweig MH, Campbell G: **Receiver-operating characteristic (ROC) plots: a fundamental evaluation tool in clinical medicine**. *Clin Chem.* 1993;**39:**561-577.

18. Mickiewicz B, Vogel HJ, Wong HR, Winston BW: **Metabolomics as a novel approach for early diagnosis of pediatric septic shock and its mortality.** *Am J Respir Crit Care Med*, 2013;**187(9):**967-976.

19. Taguchi T, Yamashita E, Mizutani T, Nakajima H, Yabuuchi M, Asano N, Miwa I: **Hepatic glycogen breakdown is implicated in the maintenance of plasma mannose concentration.** *Am J Physiol Endocrinol Metab,* 2005;**288(3):**E534-40.

20. Wills MR, Savory J: **Biochemistry of renal failure.** *Ann Clin Lab Sci*, 1981;11(4):292-299.

21. Cicarelli DD, Vieira JE, Bensenor FEM: **Comparison of C-reactive protein and serum amyloid A protein in septic shock patients.** *Mediators Inflamm,* 2008:Article ID 631414; doi:10.1155/2008/631414.

22. Dalli J, Norling LV, Montero-Melendez T, Federici Canova D, Lashin H, Pavlov AM, Sukhorukov GB, Hinds CJ, Perretti M: **Microparticle alpha-2-macroglobulin enhances pro-resolving responses and promotes survival in sepsis.** *EMBO Mol Med,* 2014;**6(1):**27-42.

23. Tian Y, Tao T, Zhu J, Zou Y, Wang J, Li J, Bo L, Deng X: **Soluble tumor necrosis factor related apoptosis inducing ligand level as a predictor of severity of sepsis and the risk of mortality in septic patients.** *PLoS One,* 2013;**8(12):**e82204.

24. Xia J, Mandal R, Sinelnikov I, Broadhurst D, Wishart DS: **MetaboAnalyst 2.0 - a comprehensive server for metabolomic data analysis.** *Nucl Acids Res,* 2012;**40:**W127-W133.

Table E1. The metabolites (non-targeted ^{1}H NMR Spectroscopy and Metabolite Concentration Profiling) and protein-mediators (targeted bead-based multiplex assay) detected and quantified.

No	Metabolites	Protein-mediators
1	2-Aminobutyrate	Interleukin (IL)-1rα
2	2-Hydroxybutyrate	IL-2
3	2-Hydroxyisovalerate	IL-4
4	2-Oxoglutarate	IL-6
5	2-Oxoisocaproate	IL-7
6	3-Hydroxybutyrate	IL-8
7	3-Hydroxyisovalerate	IL-9
8	3-Methyl-2-oxovalerate	IL-10
9	Acetate	IL-12(p70)
10	Acetoacetate	IL-17
11	Acetone	Eotaxin
12	Alanine	Fibroblast Growth Factor (FGF) basic
13	Arginine	Granulocyte Colony-Stimulating Factor (G-CSF)
14	Asparagine	Granulocyte-Macrophage Colony-Stimulating Factor (GM-CSF)
15	Aspartate	Interferon (IFN)-γ
16	Betaine	Interferon gamma-induced Protein (IP)-10
17	Carnitine	Monocyte Chemotactic Protein (MCP)-1
18	Choline	Platelet-Derived Growth Factor (PDGF)-BB
19	Citrate	Macrophage Inflammatory Protein (MIP)-1α
20	Creatine	RANTES
21	Creatine phosphate	Tumor Necrosis Factor (TNF)-α
22	Creatinine	Vascular Endothelial Growth Factor (VEGF)
23	Dimethyl sulfone	IL-2Rα
24	Dimethylamine	IL-3
25	Ethanol	IL-12p40
26	Formate	IL-16
27	Glucose	IL-18
28	Glutamate	Cutaneous T-cell-Attracting Chemokine (CTACK)
29	Glutamine	GROα
30	Glycerol	Hepatocyte Growth Factor (HGF)
31	Glycine	IFN-α2
32	Histidine	Leukemia Inhibitory Factor (LIF)
33	Isobutyrate	MCP-3
34	Isoleucine	Monocyte Colony-Stimulating Factor (M-CSF)
35	Isopropanol	Macrophage Migration Inhibitory Factor (MIF)
36	Lactate	Monokine Induced by Gamma Interferon (MIG)
37	Leucine	Stem Cell Factor (SCF)
38	Lysine	Stem Cell Growth Factor (SCGF)-β
39	Malonate	Stromal Cell-Derived Factor (SDF)-1α
40	Mannose	TNF-Related Apoptosis-Inducing Ligand (TRAIL)
41	Methanol	Procalcitonin (PCT)
42	Methionine	Ferritin

43	O-Acetylcarnitine	Tissue Plasminogen Activator (tPA)
44	O-Acetylcholine	Serum Amyloid A (SAA)
45	Ornithine	Alpha 2-Macroglobulin (A2M)
46	Phenylalanine	Haptoglobin
47	Proline	C-Reactive Protein (CRP)
48	Propylene glycol	Serum Amyloid P (SAP)
49	Pyruvate	
50	Serine	
51	Taurine	
52	Threonine	
53	Trimethylamine N-oxide	
54	Tyrosine	
55	Urea	
56	Valine	
57	cis-Aconitate	
58	myo-Inositol	

TableE2. Comparison of statistical measures calculated for the supervised OPLS-DA models without excluding outliers to the results presented in the manuscript.

**Data**	**PLS-DA model All cohorts**		**OPLS-DA model PICU-sepsis versus ED-sepsis**	
	**Outliers included**	**Outliers excluded (see manuscript)**	**Outliers included**	**Outliers excluded (see manuscript)**
Metabolomics data	$R2Y_{cum}$ = 0.56<br>$Q2_{cum}$ = 0.49	$R2Y_{cum}$ = 0.56<br>$Q2_{cum}$ = 0.47	R2Y = 0.69<br>Q2 = 0.63	R2Y = 0.69<br>Q2 = 0.60
Protein-mediators data	$R2Y_{cum}$ = 0.53<br>$Q2_{cum}$ = 0.45	$R2Y_{cum}$ = 0.54<br>$Q2_{cum}$ = 0.47	R2Y = 0.54<br>Q2 = 0.47	R2Y = 0.58<br>Q2 = 0.42
Combined dataset	$R2Y_{cum}$ = 0.63<br>$Q2_{cum}$ = 0.57	$R2Y_{cum}$ = 0.63<br>$Q2_{cum}$ = 0.56	R2Y = 0.67<br>Q2 = 0.63	R2Y = 0.69<br>Q2 = 0.62
