# Supplementary material for: Development of metabolic and inflammatory mediator biomarker phenotyping for early diagnosis and triage of pediatric sepsis
Source: Crit Care. 2015 Sep 9;19(1):320. doi: 10.1186/s13054-015-1026-2 (PMC4563828; doi:10.1186/s13054-015-1026-2)
Supplement: Additional file 2: — Results of the metabolomic and protein mediator biomarker phenotyping in the two age subgroups. This file contains six figures depicting the results for the age subgroups and a seventh figure showing the loading plots that demonstrate which metabolites and/or inflammatory protein mediators contribute most to each component in the PCA models for the age 2–17-year-old cohort. (PDF 4020 kb) [file 13054_2015_1026_MOESM2_ESM.pdf]

Early diagnosis and triage of pediatric sepsis using metabolic and inflammatory mediator biomarkers.

Authors: Beata Mickiewicz PhD, Graham Thompson MD, Jaime Blackwood MD, Craig N Jenne PhD, Brent W Winston MD, Hans J Vogel PhD, Ari R Joffe MD, for the Alberta Sepsis Network.
Journal: Critical Care
Corresponding Author: Ari R Joffe MD; University of Alberta, Edmonton, Alberta, Canada; email: ari.joffe@albertahealthservices.ca

Additional File 2: Results of the metabolomic and protein-mediator biomarker phenotyping in the two age subgroups.

Fig. E1 Principal Component Analysis results for the 2-5 year old cohorts, using metabolomics (a), protein-mediators (b), and combined metabolomics and protein-mediator (c) data. Red circles: PICU-sepsis cohort; Blue circles: ED-sepsis cohort; Green circles: ED-control cohort.

Fig. E2 Partial Least Squares Discriminant Analysis for the 2-5 year old cohorts, using metabolomics (a), protein-mediators (b), and combined metabolomics and protein-mediator (c) data. Red circles: PICU-sepsis cohort; Blue circles: ED-sepsis cohort; Green circles: ED-control cohort.

Fig. E3 Orthogonal Partial Least Squares Discriminant Analysis for the 2-5 year old cohorts, using metabolomics (a), protein-mediator (b), and combined metabolomics and protein-mediator (c) data. Red circles: PICU-sepsis cohort; Blue circles: ED-sepsis cohort; Green circles: ED-control cohort.

Fig. E4 Principal Component Analysis results for the 6-17 year old cohorts, using metabolomics (a), protein-mediator (b), and combined metabolomics and protein-mediator (c) data. Red circles: PICU-sepsis cohort; Blue circles: ED-sepsis cohort; Green circles: ED-control cohort.

Fig. E5 Partial Least Squares Discriminant Analysis for the 6-17 year old cohorts, using metabolomics (a), protein-mediator (b), and combined metabolomics and protein-mediator (c) data. Red circles: PICU-sepsis cohort; Blue circles: ED-sepsis cohort; Green circles: ED-control cohort.

Fig. E6 Orthogonal Partial Least Squares Discriminant Analysis for the 6-17 year old cohorts, using metabolomics (a), protein-mediator (b), and combined metabolomics and protein-mediator (c) data. Red circles: PICU-sepsis cohort; Blue circles: ED-sepsis cohort; Green circles: ED-control cohort.

Fig. E7 The loading plots for each principal component (PC1, PC2 and PC3) for PCA models calculated for the 2-17 year old cohorts using metabolic profiling (a), inflammatory protein-mediator profiling (b), and combined biomarker profiling (c) data. The loadings plots demonstrate which metabolites/inflammatory protein-mediators most contribute to each component in the PCA models (the bigger bar, the more influential the variable) and how these variables are correlated.

Figure E1. PCA Results for 2-5 year old cohorts.

a. Metabolomics results

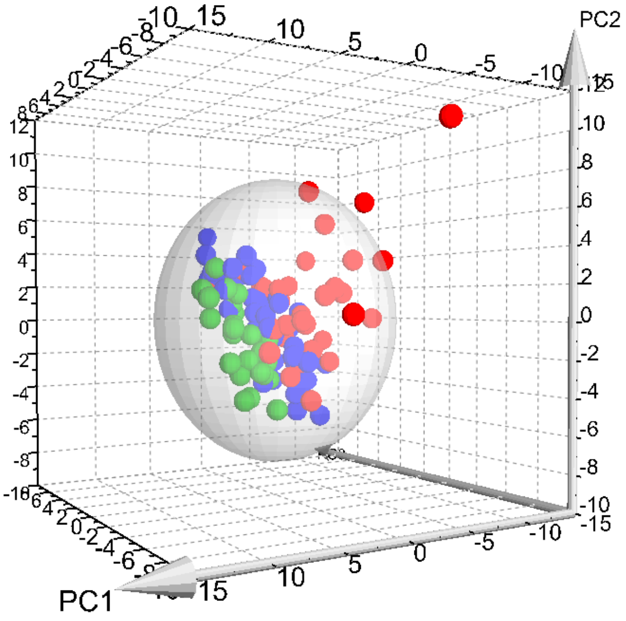

$R2X_{cum} = 0.426$;
$Q2_{cum} = 0.261$

PC1 = 19.7%
PC2 = 14.5%
PC3 = 8.4%

b. Protein-mediator results

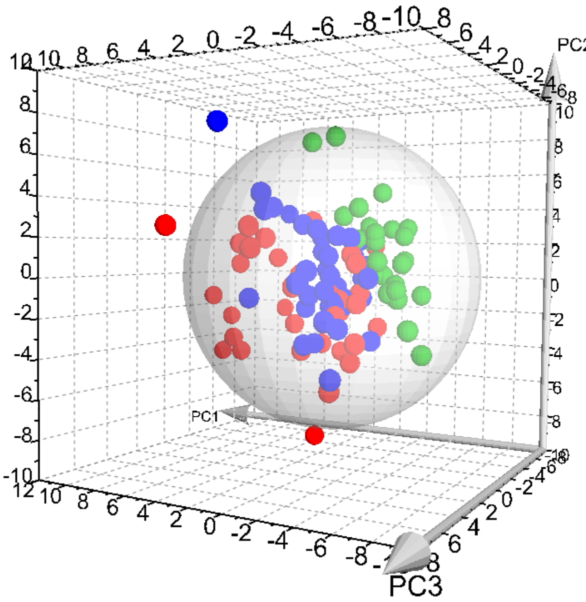

$R2X_{cum} = 0.44$;
$Q2_{cum} = 0.257$

PC1 = 19.9%
PC2 = 15.9%
PC3 = 8.26%

c. Combined results.

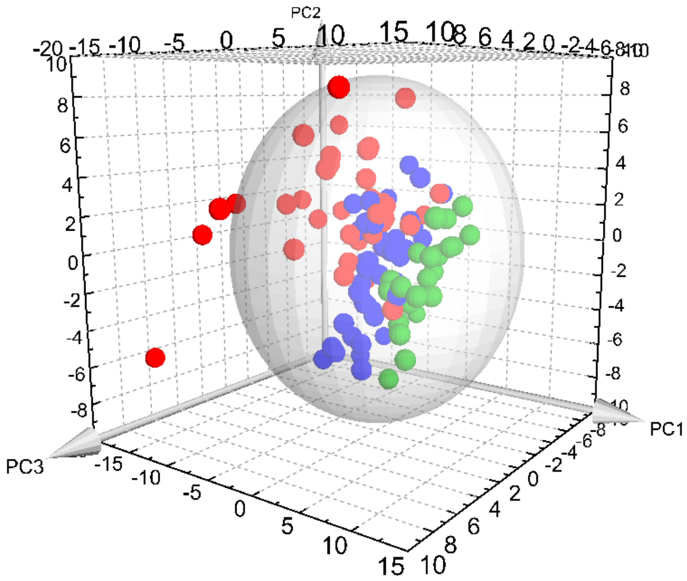

$R2X_{cum}$ = 0.383;
$Q2_{cum}$ = 0.261

PC1 = 21.7%
PC2 = 9.72%
PC3 = 6.87%

Figure E2. PLS-DA results for 2-5 year old cohort.

a. Metabolomics results

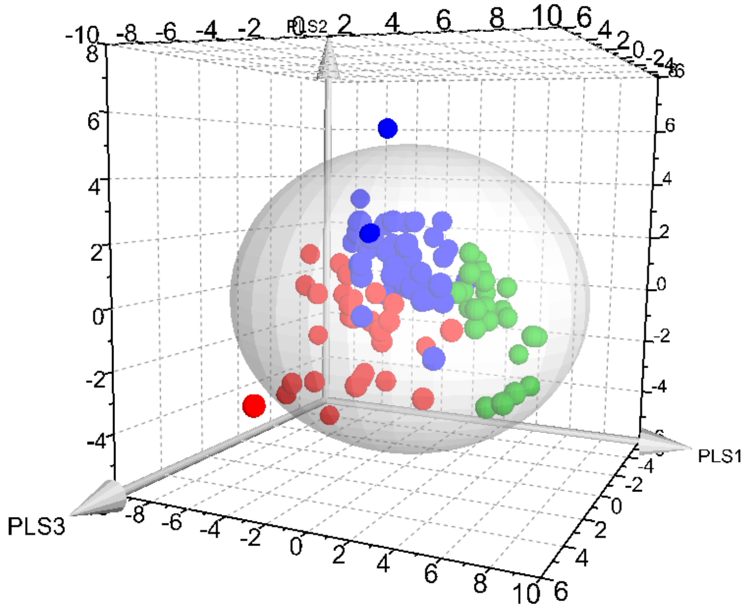

R2Y$_{cum}$ = 0.61;
Q2$_{cum}$ = 0.43

b. Protein-mediator results

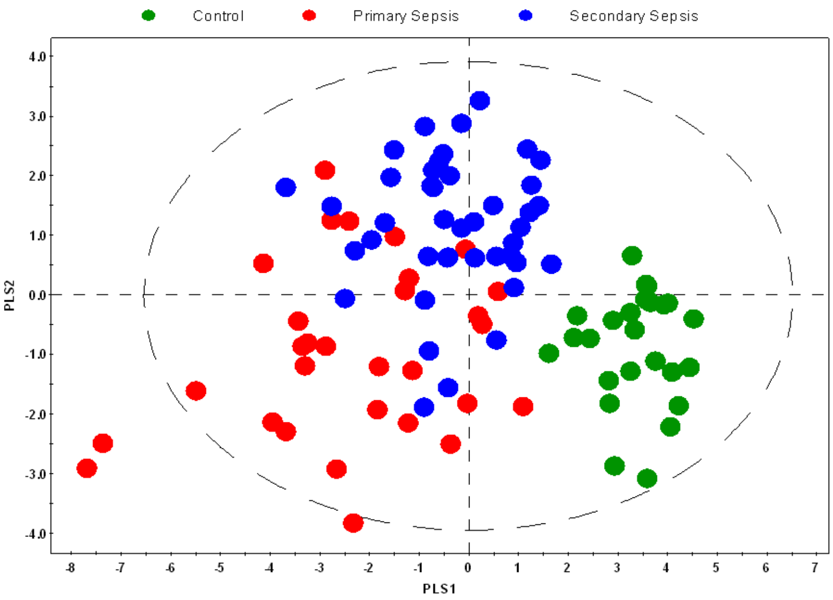

R2Y$_{cum}$ = 0.55
Q2$_{cum}$ = 0.36

c. Combined results

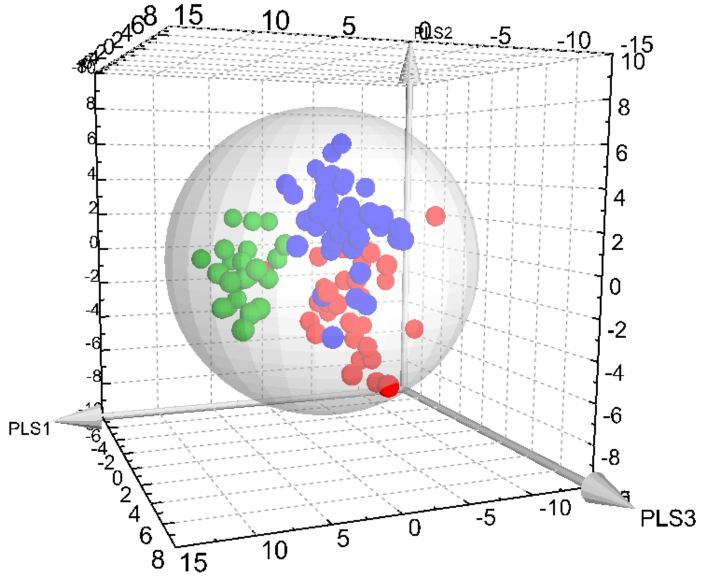

$R2Y_{cum}$ = 0.69;
$Q2_{cum}$ = 0.57

Figure E3. OPLS-DA results for the 2-5 year old cohorts.

a. Metabolomics

PICU-sepsis vs. ED-sepsis

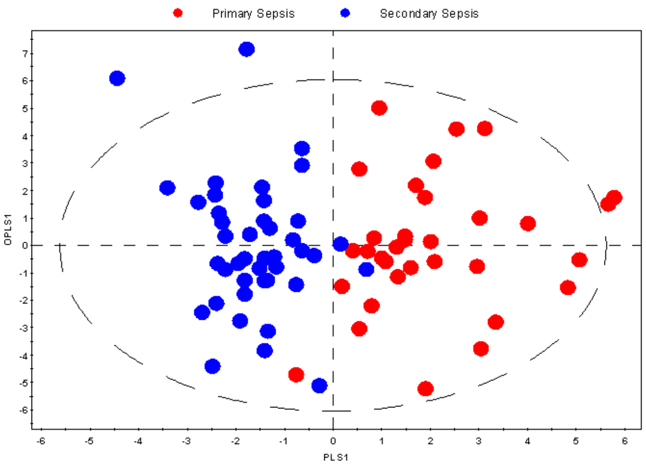

R2Y = 0.68; Q2 = 0.50

ED-sepsis vs. ED-controls

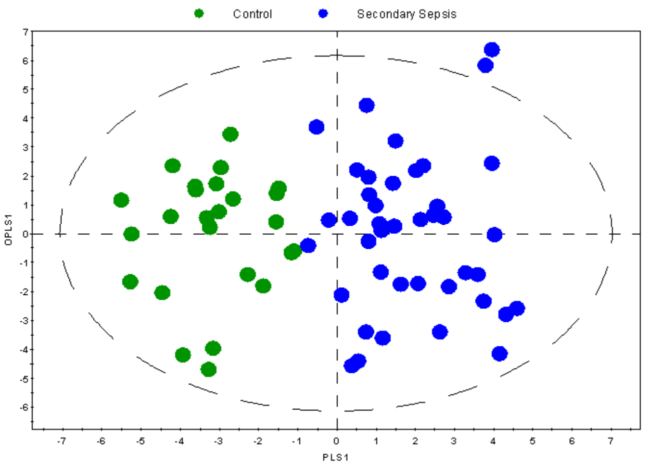

R2Y = 0.76; Q2 = 0.66

PICU-sepsis vs. ED-controls

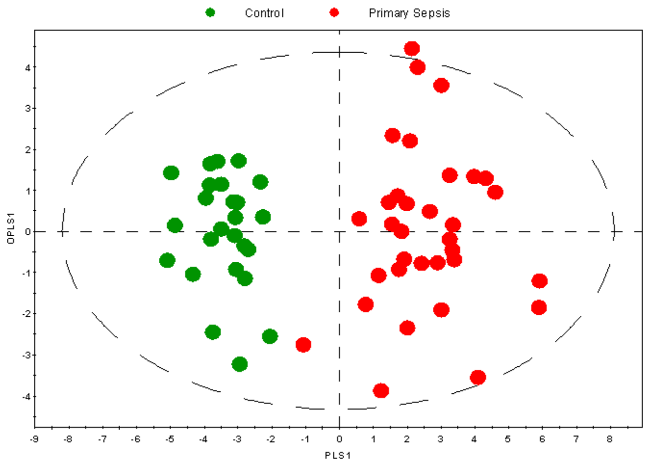

R2Y = 0.86; Q2 = 0.73

b. Protein-mediators

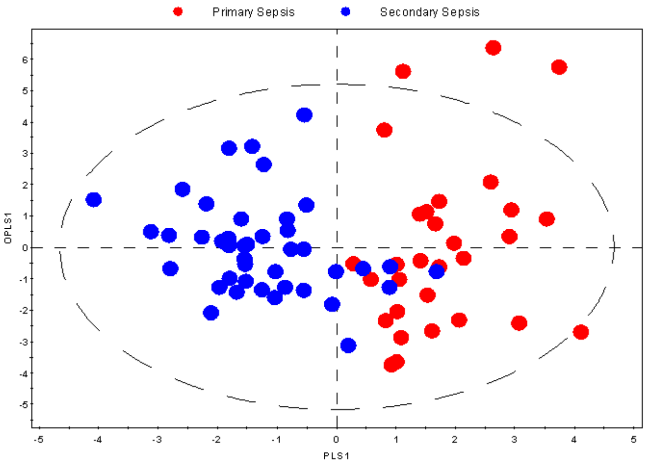

R2Y = 0.67; Q2 = 0.45

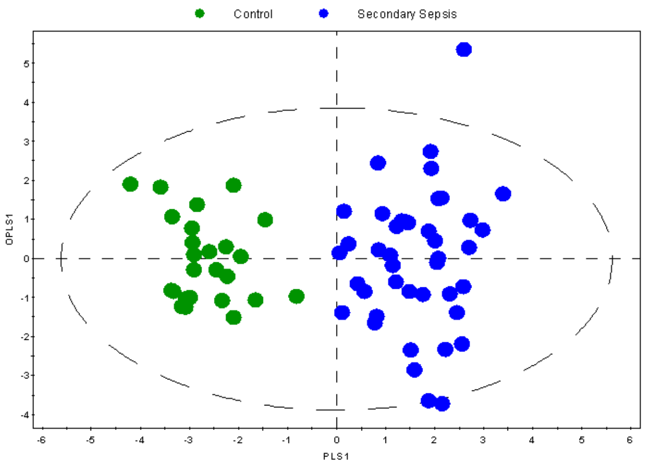

R2Y = 0.87; Q2 = 0.82

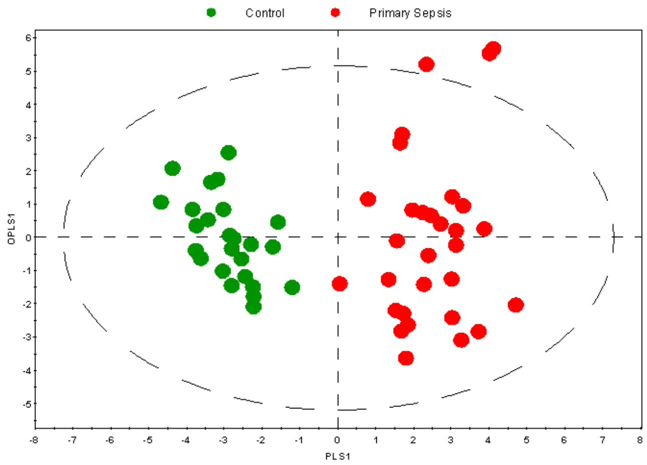

R2Y = 0.89; Q2 = 0.82

c. Combined results

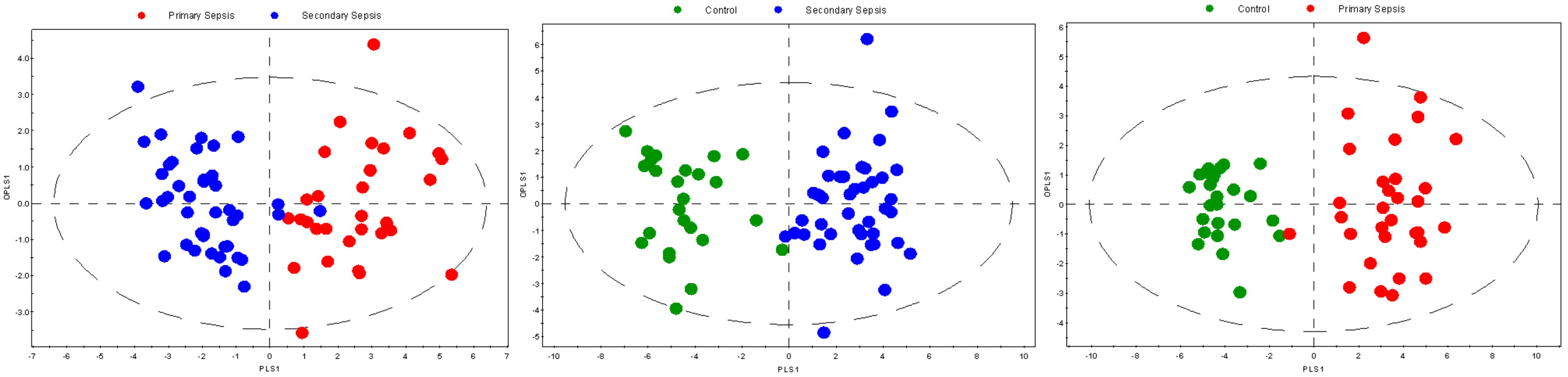

R2Y = 0.78; Q2 = 0.65 R2Y = 0.86; Q2 = 0.82 R2Y = 0.88; Q2 = 0.84

Figure E4. PCA Results for 6-17 year old cohorts.

a. Metabolomics results

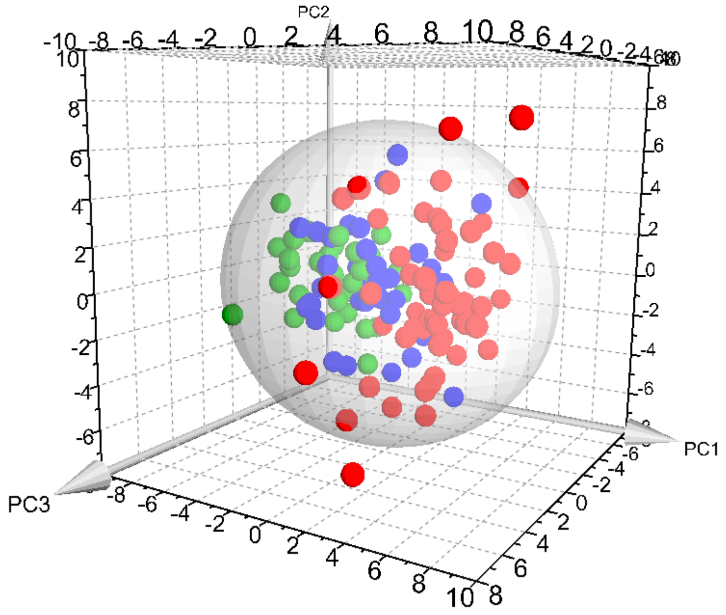

$R2X_{cum}$ = 0.386;
$Q2_{cum}$ = 0.24

PC1 = 18.5%
PC2 = 12.5%
PC3 = 7.65%

b. Protein-mediator results

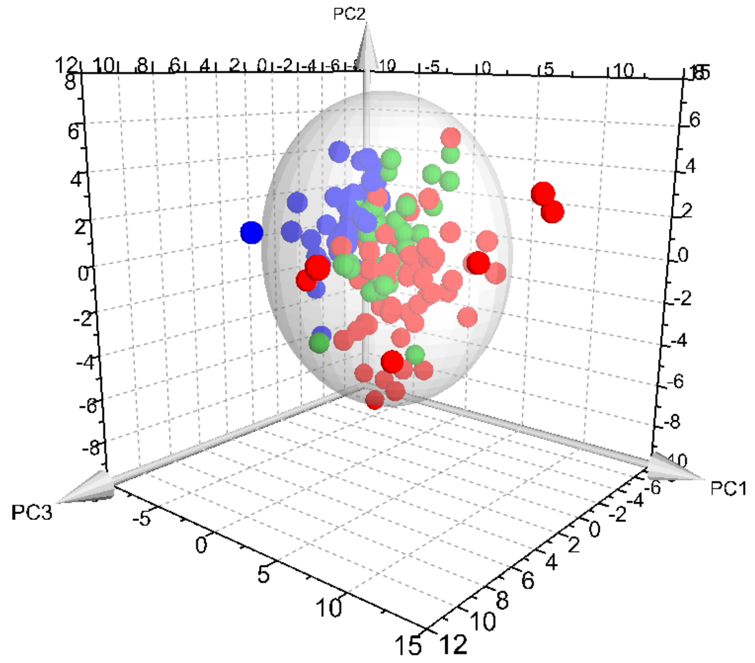

$R2X_{cum}$ = 0.425;
$Q2_{cum}$ = 0.233

PC1 = 19.9%
PC2 = 14%
PC3 = 8.65%

c. Combined results.

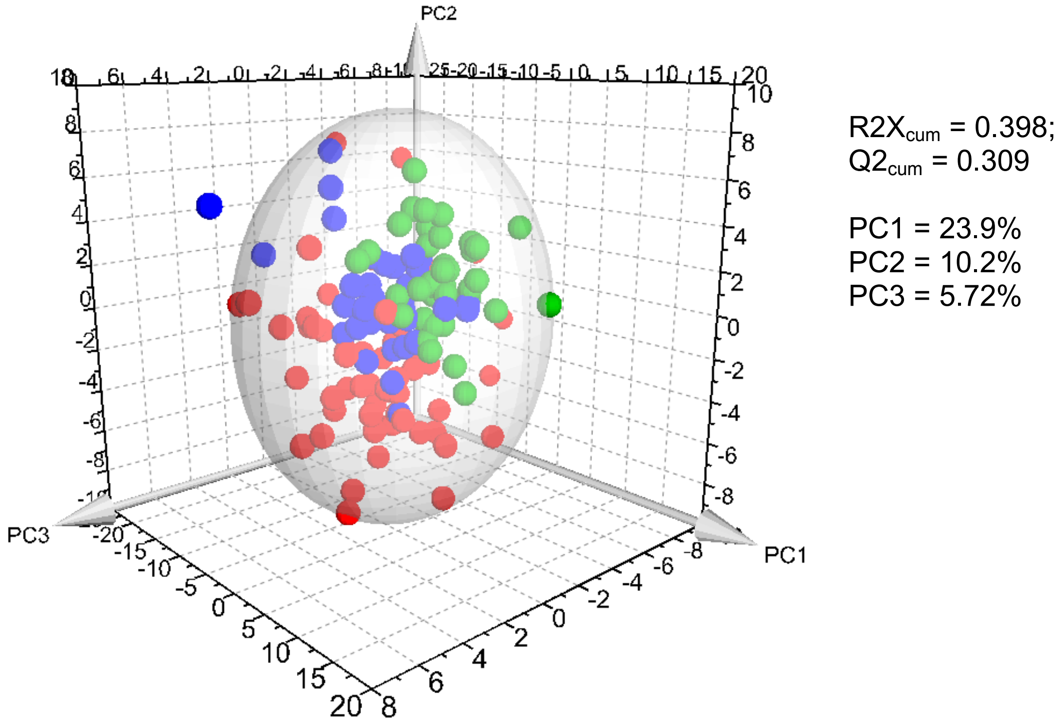

Figure E5. PLS-DA results for 6-17 year old cohort.

a. Metabolomics results

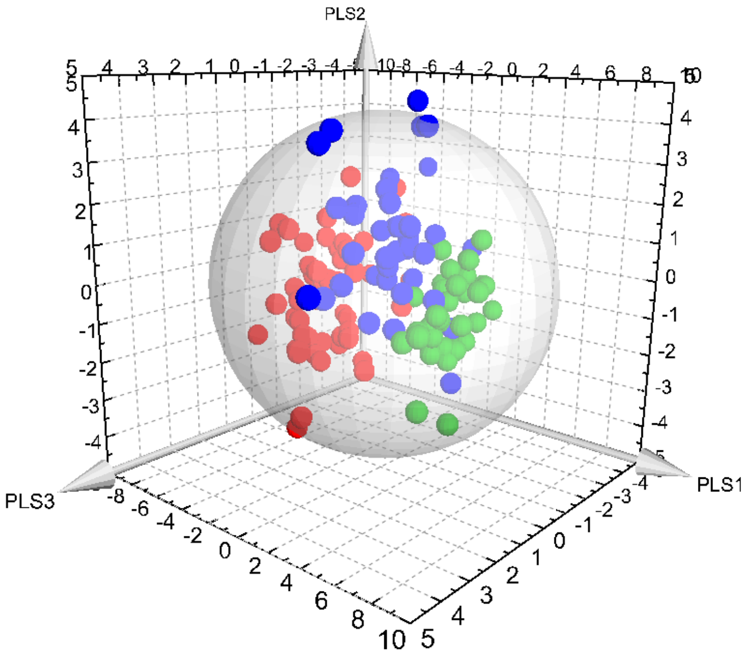

$R2Y_{cum} = 0.62$;
$Q2_{cum} = 0.42$

b. Protein-mediator results

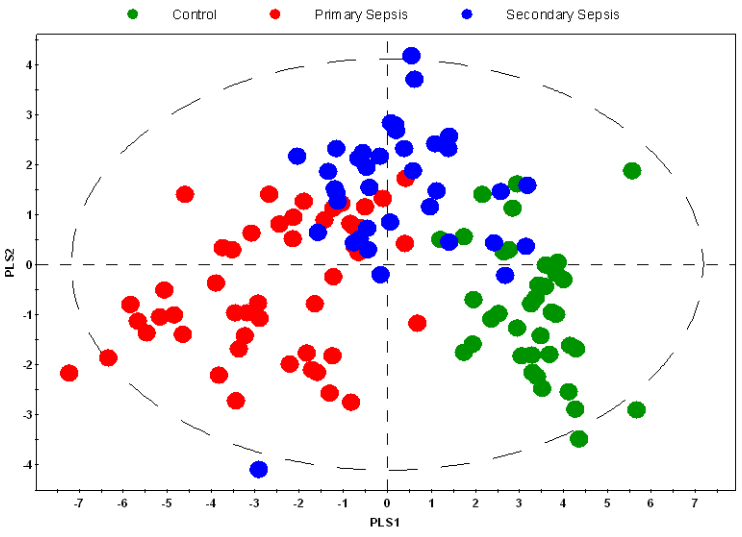

$R2Y_{cum} = 0.54$;
$Q2_{cum} = 0.45$

c. Combined results

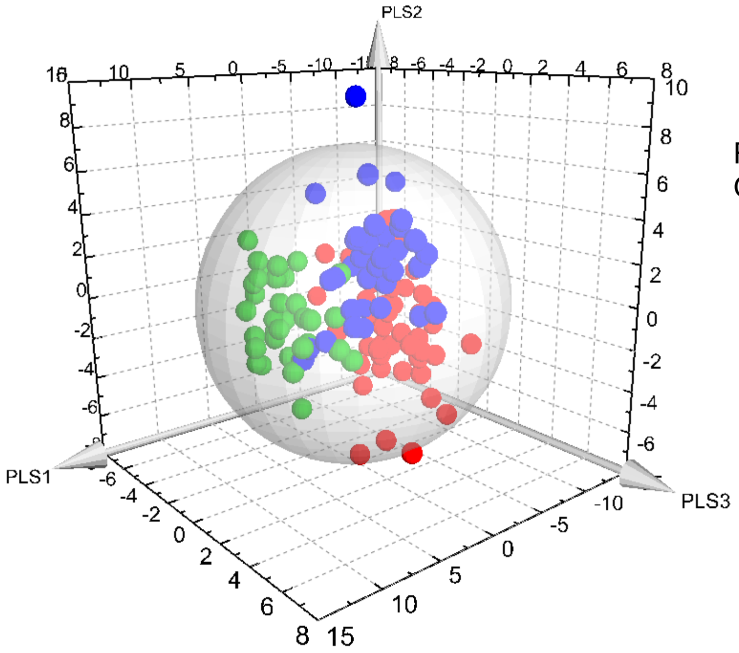

$R2Y_{cum} = 0.70$;
$Q2_{cum} = 0.61$

Figure E6. OPLS-DA results for the 6-17 year old cohorts.

a. Metabolomics

PICU-sepsis vs. ED-sepsis

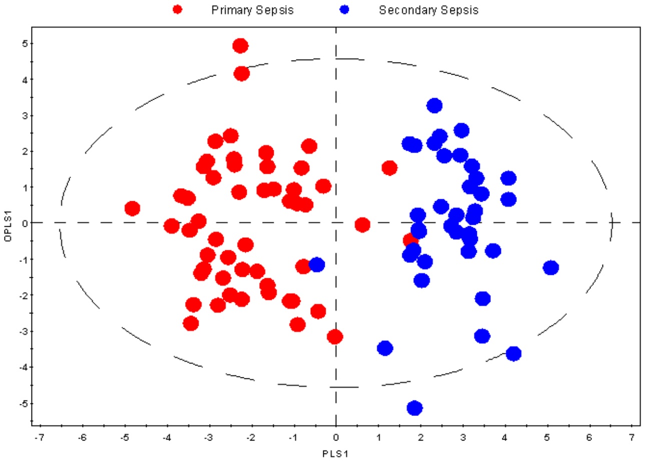

R2Y = 0.79; Q2 = 0.68

ED-sepsis vs. ED-controls

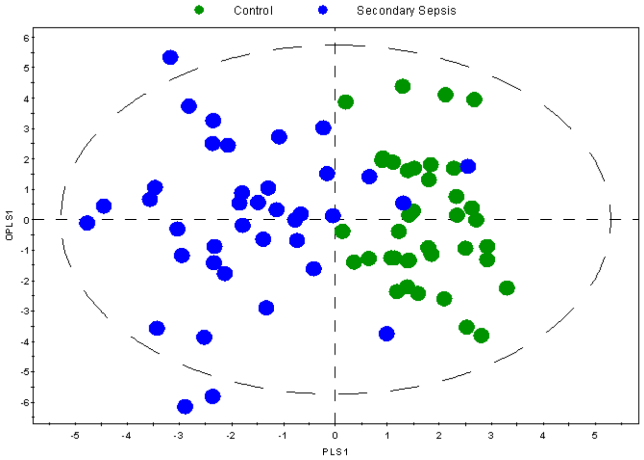

R2Y = 0.66; Q2 = 0.45

PICU-sepsis vs. ED-controls

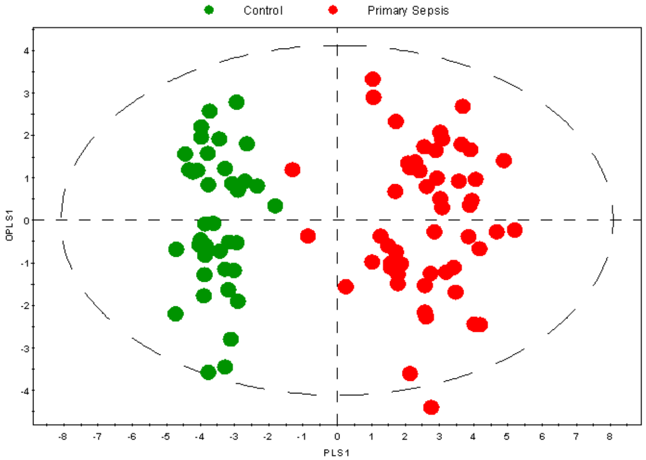

R2Y = 0.89; Q2 = 0.83

b. Protein-mediators

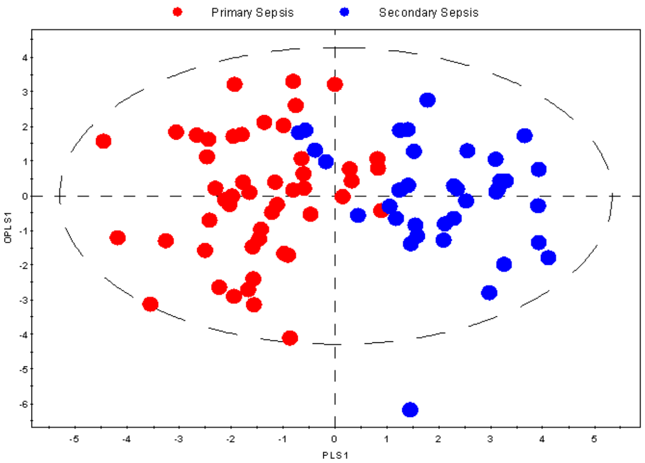

R2Y = 0.67; Q2 = 0.45

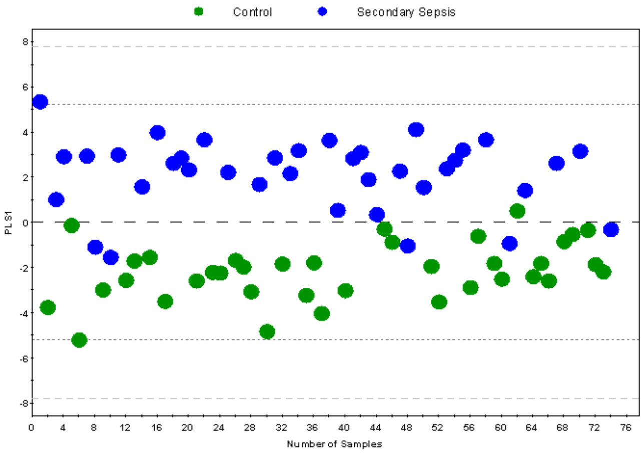

R2Y = 0.70; Q2 = 0.64

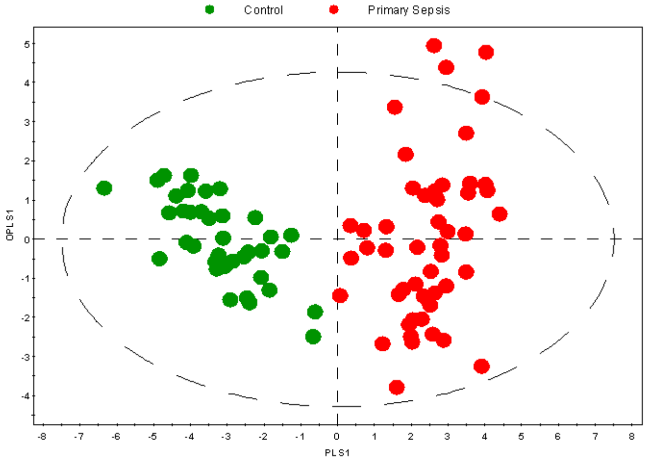

R2Y = 0.86; Q2 = 0.79

c. Combined results

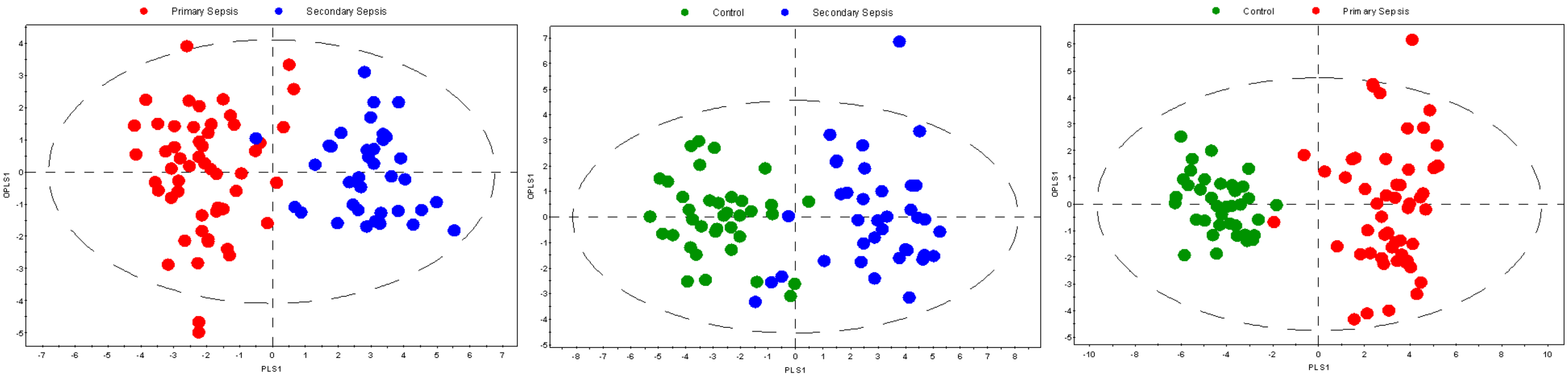

R2Y = 0.82; Q2 = 0.76

R2Y = 0.77; Q2 = 0.70

R2Y = 0.88; Q2 = 0.86

Fig. E7 The loading plots for each principal component (PC1, PC2 and PC3) for PCA models in 2-17y cohort.

a. Metabolic profiling data

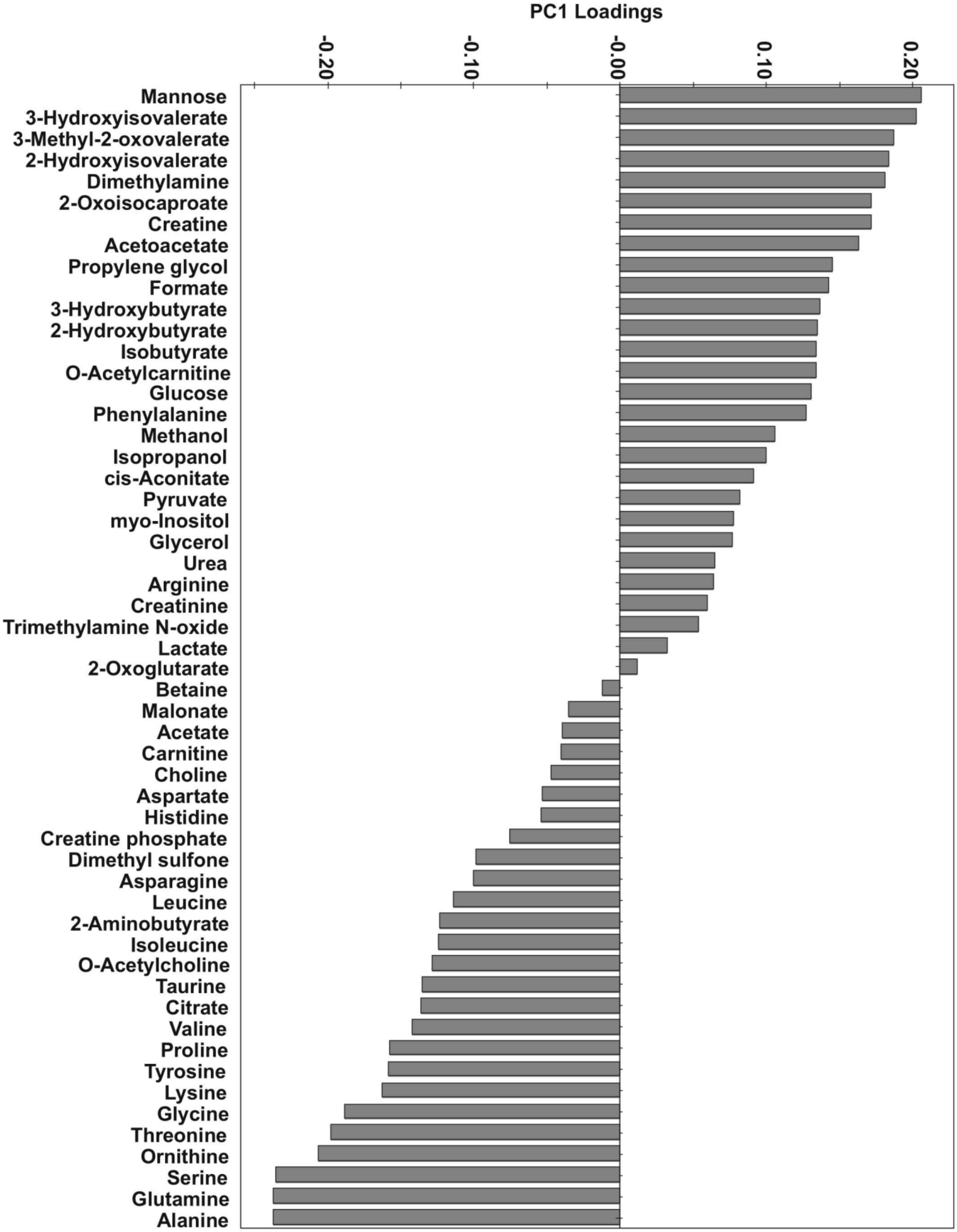

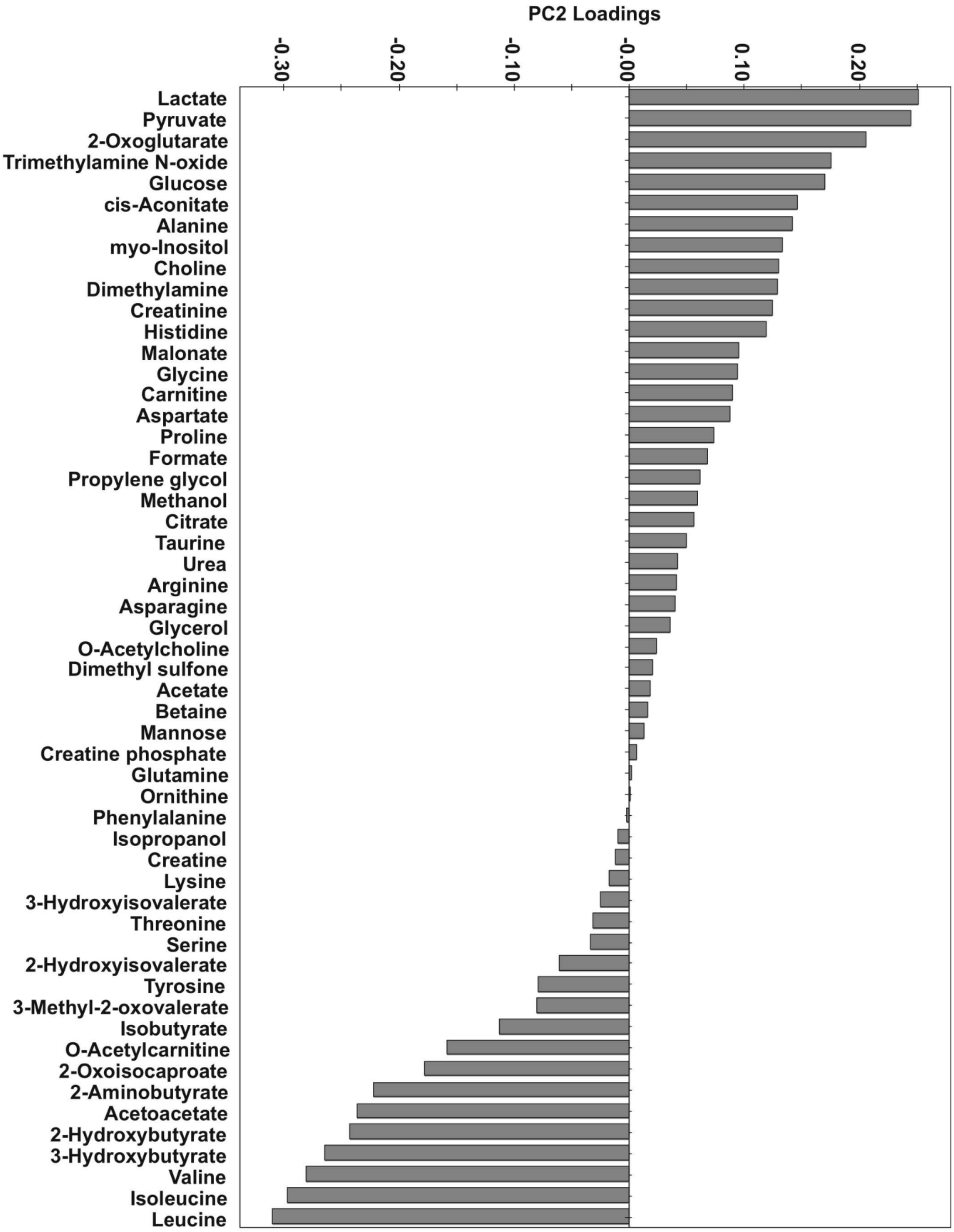
PC2 Loadings
-0.30
-0.20
-0.10
-0.00
0.10
0.20
Lactate
Pyruvate
2-Oxoglutarate
Trimethylamine N-oxide
Glucose
cis-Aconitate
Alanine
myo-Inositol
Choline
Dimethylamine
Creatinine
Histidine
Malonate
Glycine
Carnitine
Aspartate
Proline
Formate
Propylene glycol
Methanol
Citrate
Taurine
Urea
Arginine
Asparagine
Glycerol
O-Acetylcholine
Dimethyl sulfone
Acetate
Betaine
Mannose
Creatine phosphate
Glutamine
Ornithine
Phenylalanine
Isopropanol
Creatine
Lysine
3-Hydroxyisovalerate
Threonine
Serine
2-Hydroxyisovalerate
Tyrosine
3-Methyl-2-oxovalerate
Isobutyrate
O-Acetylcarnitine
2-Oxoisocaproate
2-Aminobutyrate
Acetoacetate
2-Hydroxybutyrate
3-Hydroxybutyrate
Valine
Isoleucine
Leucine

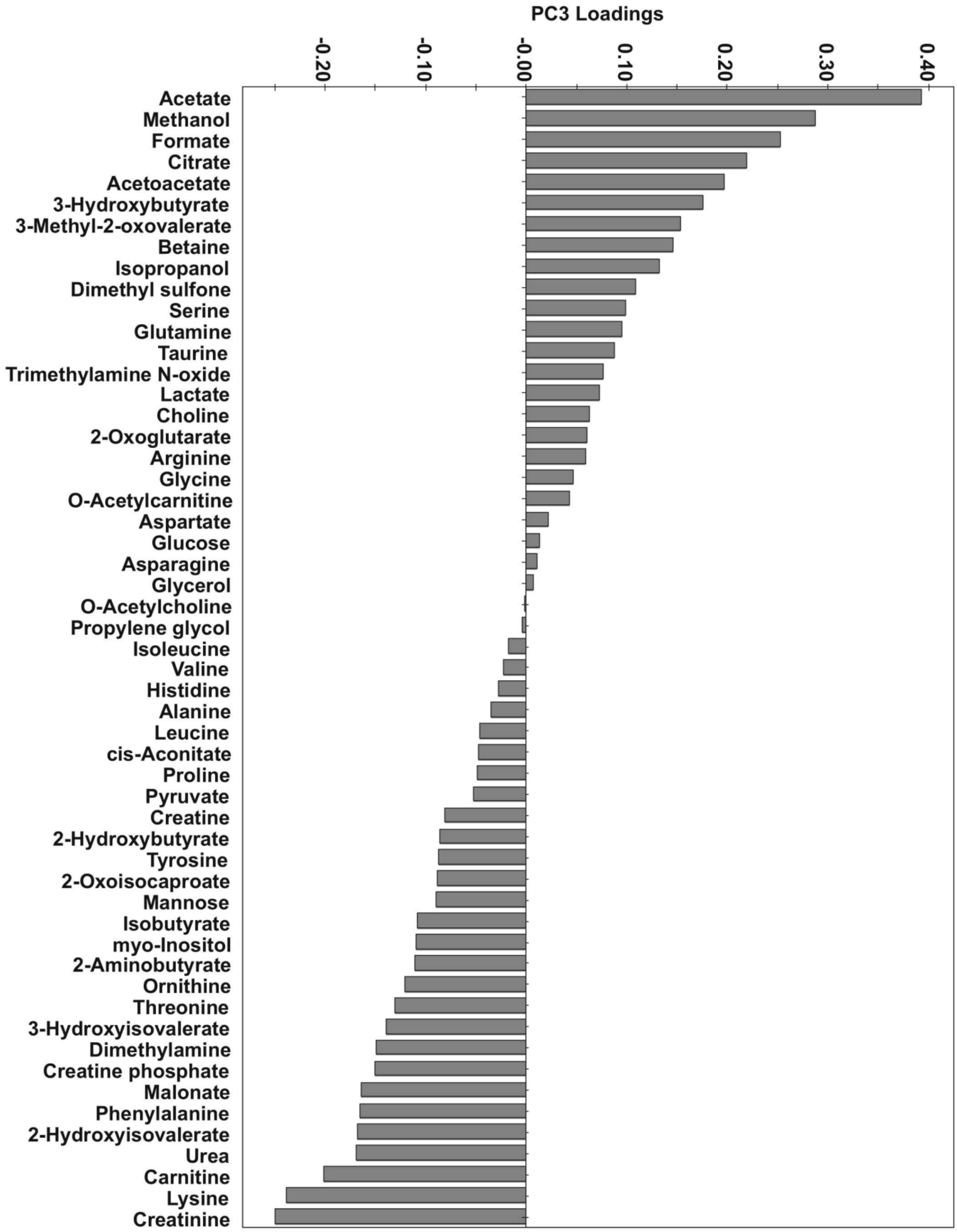
PC3 Loadings
-0.20
-0.10
-0.00
0.10
0.20
0.30
0.40
Acetate
Methanol
Formate
Citrate
Acetoacetate
3-Hydroxybutyrate
3-Methyl-2-oxovalerate
Betaine
Isopropanol
Dimethyl sulfone
Serine
Glutamine
Taurine
Trimethylamine N-oxide
Lactate
Choline
2-Oxoglutarate
Arginine
Glycine
O-Acetylcarnitine
Aspartate
Glucose
Asparagine
Glycerol
O-Acetylcholine
Propylene glycol
Isoleucine
Valine
Histidine
Alanine
Leucine
cis-Aconitate
Proline
Pyruvate
Creatine
2-Hydroxybutyrate
Tyrosine
2-Oxoisocaproate
Mannose
Isobutyrate
myo-Inositol
2-Aminobutyrate
Ornithine
Threonine
3-Hydroxyisovalerate
Dimethylamine
Creatine phosphate
Malonate
Phenylalanine
2-Hydroxyisovalerate
Urea
Carnitine
Lysine
Creatinine

b. Inflammatory protein-mediator profiling results

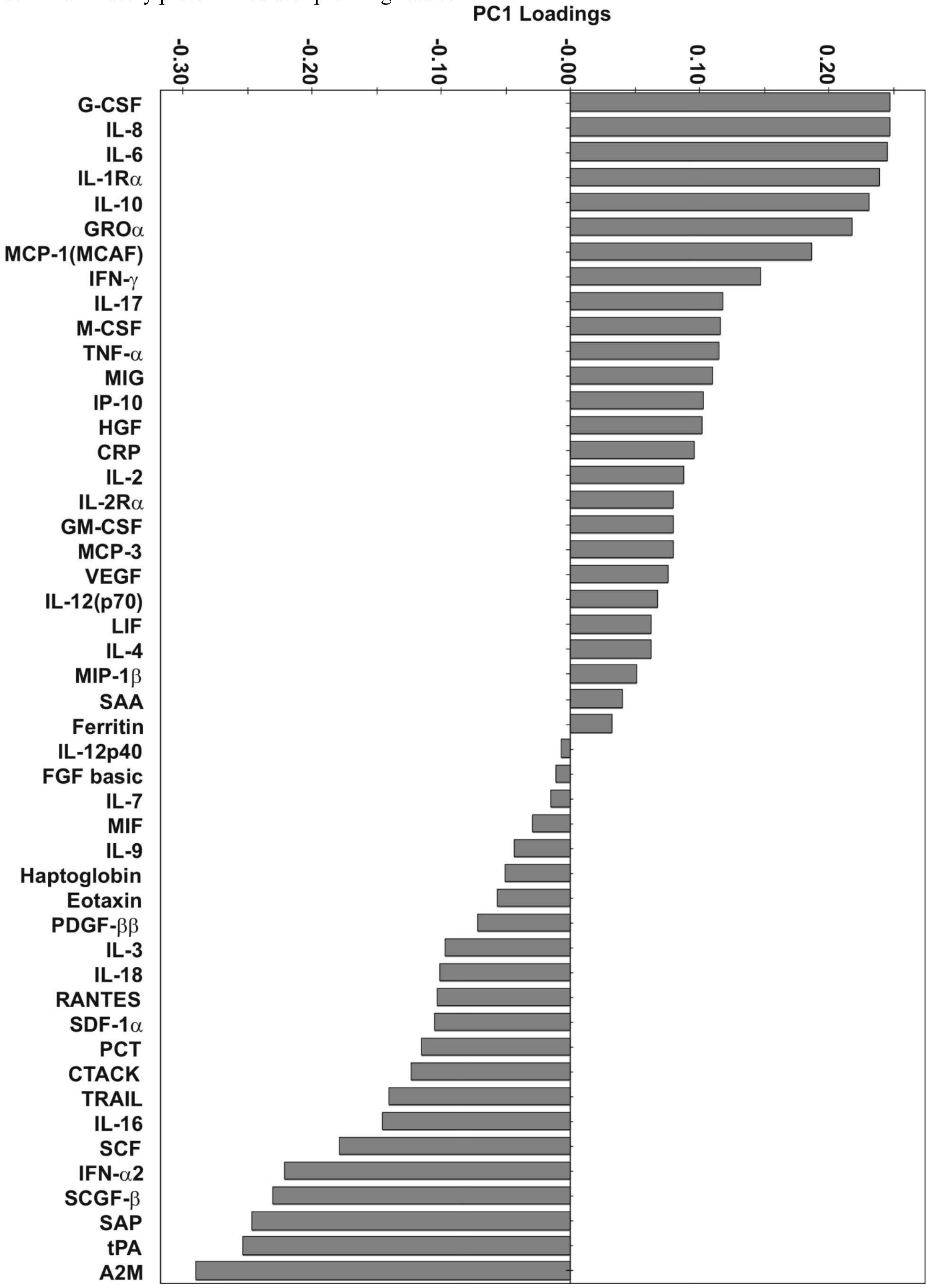

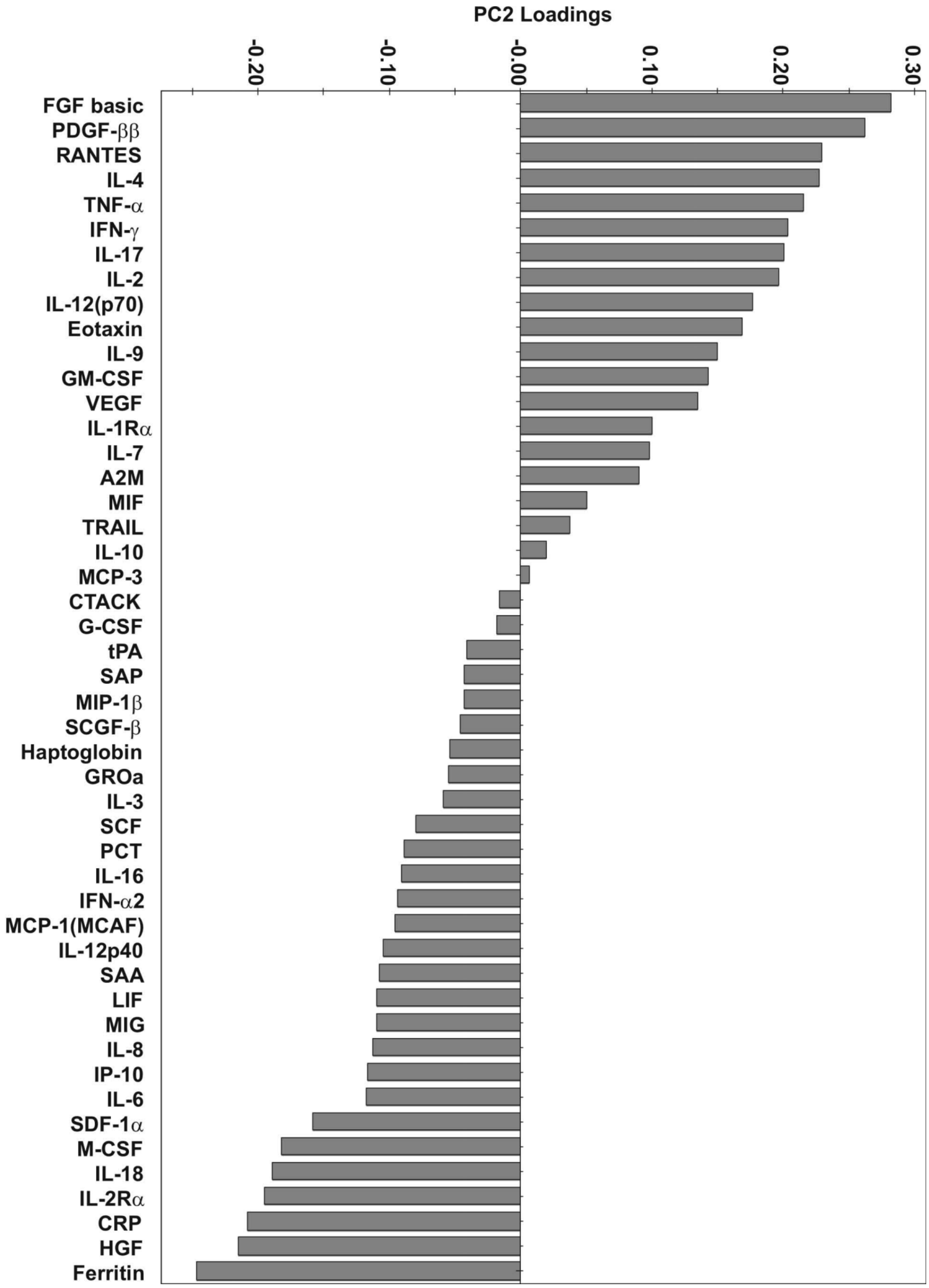
PC2 Loadings
-0.20
-0.10
-0.00
0.10
0.20
0.30
FGF basic
PDGF-ββ
RANTES
IL-4
TNF-α
IFN-γ
IL-17
IL-2
IL-12(p70)
Eotaxin
IL-9
GM-CSF
VEGF
IL-1Rα
IL-7
A2M
MIF
TRAIL
IL-10
MCP-3
CTACK
G-CSF
tPA
SAP
MIP-1β
SCGF-β
Haptoglobin
GROa
IL-3
SCF
PCT
IL-16
IFN-α2
MCP-1(MCAF)
IL-12p40
SAA
LIF
MIG
IL-8
IP-10
IL-6
SDF-1α
M-CSF
IL-18
IL-2Rα
CRP
HGF
Ferritin

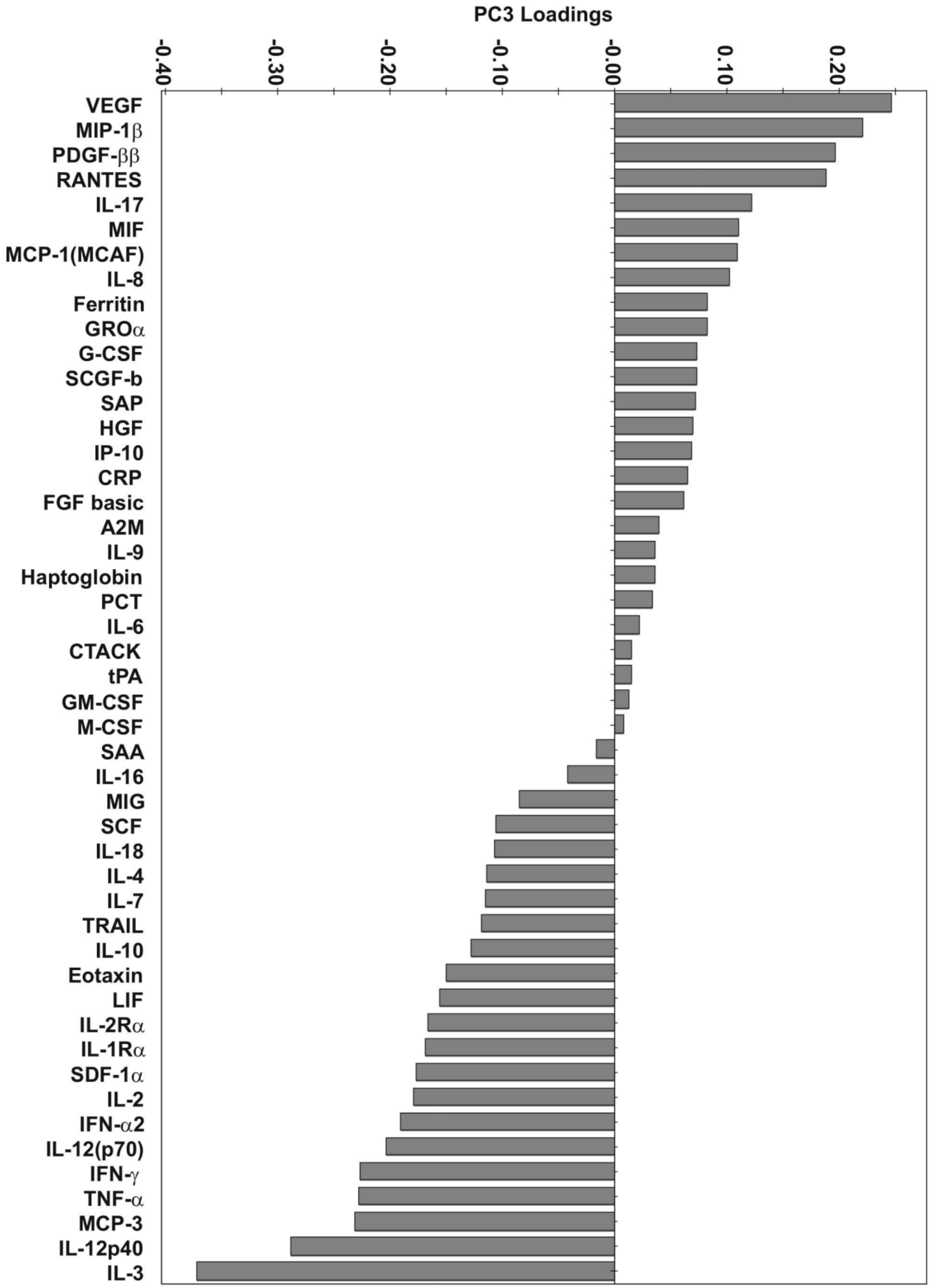
PC3 Loadings
-0.40
-0.30
-0.20
-0.10
-0.00
0.10
0.20
VEGF
MIP-1β
PDGF-ββ
RANTES
IL-17
MIF
MCP-1(MCAF)
IL-8
Ferritin
GROα
G-CSF
SCGF-b
SAP
HGF
IP-10
CRP
FGF basic
A2M
IL-9
Haptoglobin
PCT
IL-6
CTACK
tPA
GM-CSF
M-CSF
SAA
IL-16
MIG
SCF
IL-18
IL-4
IL-7
TRAIL
IL-10
Eotaxin
LIF
IL-2Rα
IL-1Rα
SDF-1α
IL-2
IFN-α2
IL-12(p70)
IFN-γ
TNF-α
MCP-3
IL-12p40
IL-3

c. Combined Results

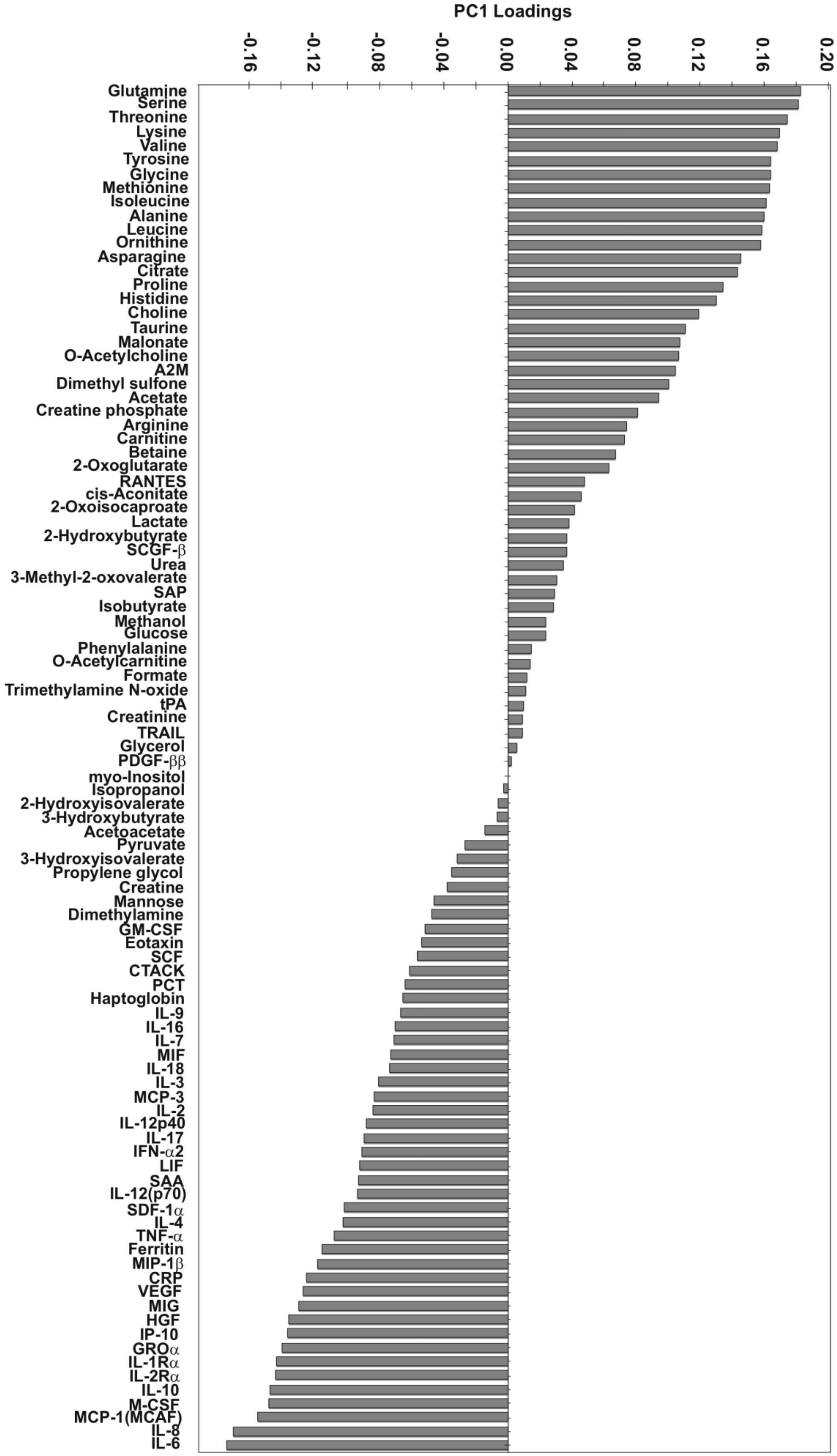

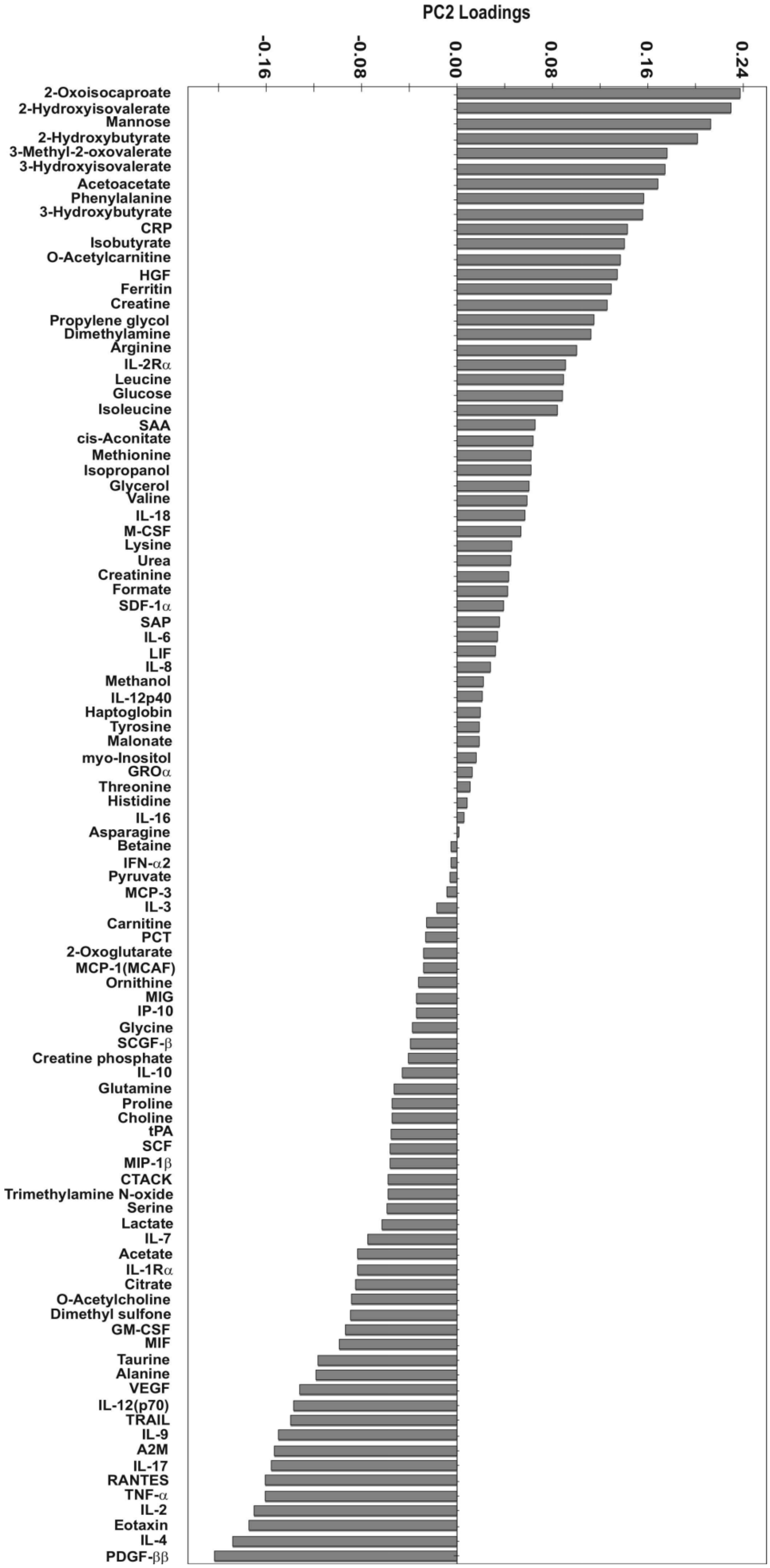
PC2 Loadings
-0.16
-0.08
0.00
0.08
0.16
0.24
2-Oxoisocaproate
2-Hydroxyisovalerate
Mannose
2-Hydroxybutyrate
3-Methyl-2-oxovalerate
3-Hydroxyisovalerate
Acetoacetate
Phenylalanine
3-Hydroxybutyrate
CRP
Isobutyrate
O-Acetylcarnitine
HGF
Ferritin
Creatine
Propylene glycol
Dimethylamine
Arginine
IL-2Rα
Leucine
Glucose
Isoleucine
SAA
cis-Aconitate
Methionine
Isopropanol
Glycerol
Valine
IL-18
M-CSF
Lysine
Urea
Creatinine
Formate
SDF-1α
SAP
IL-6
LIF
IL-8
Methanol
IL-12p40
Haptoglobin
Tyrosine
Malonate
myo-Inositol
GROα
Threonine
Histidine
IL-16
Asparagine
Betaine
IFN-α2
Pyruvate
MCP-3
IL-3
Carnitine
PCT
2-Oxoglutarate
MCP-1(MCAF)
Ornithine
MIG
IP-10
Glycine
SCGF-β
Creatine phosphate
IL-10
Glutamine
Proline
Choline
tPA
SCF
MIP-1β
CTACK
Trimethylamine N-oxide
Serine
Lactate
IL-7
Acetate
IL-1Rα
Citrate
O-Acetylcholine
Dimethyl sulfone
GM-CSF
MIF
Taurine
Alanine
VEGF
IL-12(p70)
TRAIL
IL-9
A2M
IL-17
RANTES
TNF-α
IL-2
Eotaxin
IL-4
PDGF-ββ

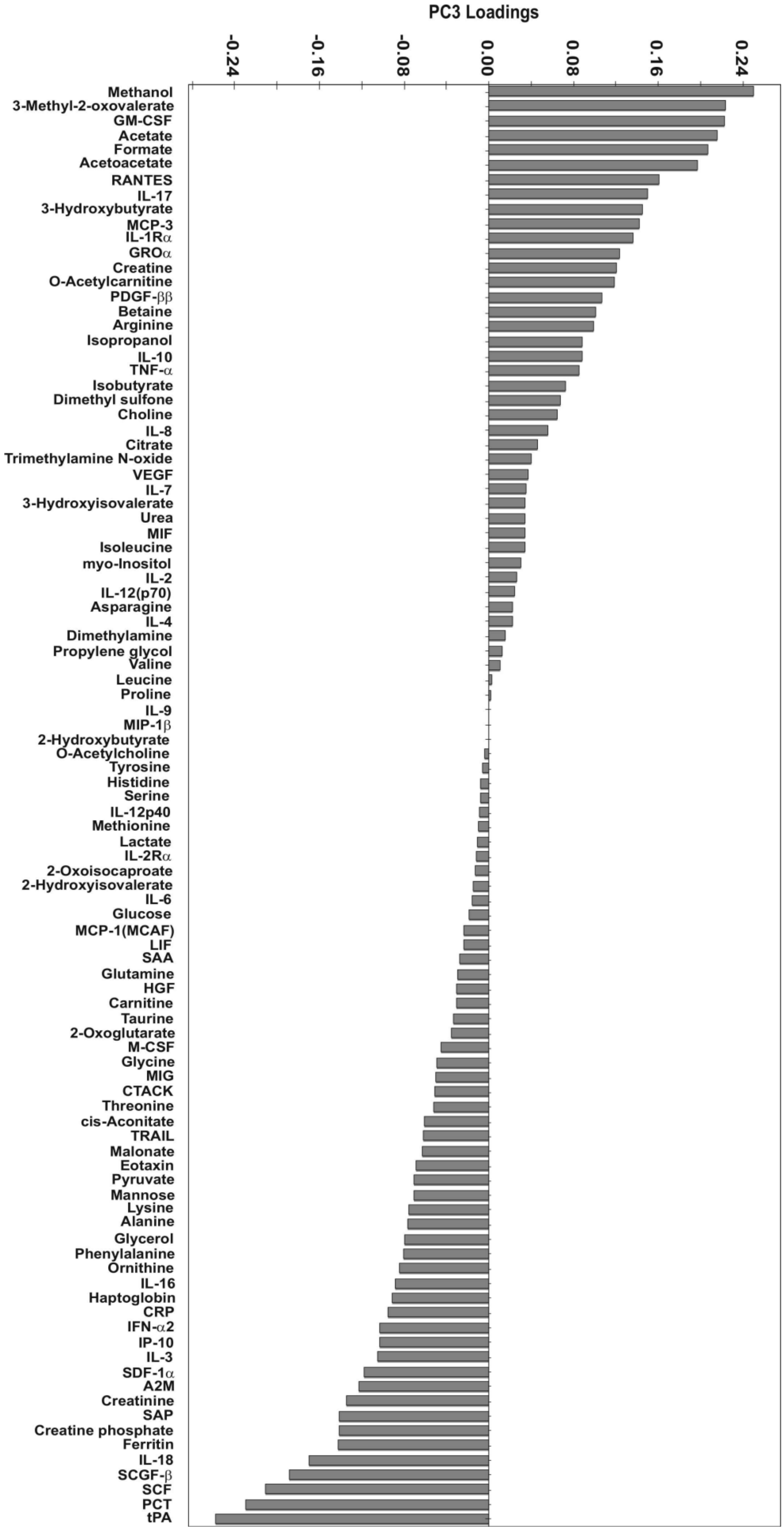
PC3 Loadings
-0.24
-0.16
-0.08
0.00
0.08
0.16
0.24
Methanol
3-Methyl-2-oxovalerate
GM-CSF
Acetate
Formate
Acetoacetate
RANTES
IL-17
3-Hydroxybutyrate
MCP-3
IL-1Rα
GROα
Creatine
O-Acetylcarnitine
PDGF-ββ
Betaine
Arginine
Isopropanol
IL-10
TNF-α
Isobutyrate
Dimethyl sulfone
Choline
IL-8
Citrate
Trimethylamine N-oxide
VEGF
IL-7
3-Hydroxyisovalerate
Urea
MIF
Isoleucine
myo-Inositol
IL-2
IL-12(p70)
Asparagine
IL-4
Dimethylamine
Propylene glycol
Valine
Leucine
Proline
IL-9
MIP-1β
2-Hydroxybutyrate
O-Acetylcholine
Tyrosine
Histidine
Serine
IL-12p40
Methionine
Lactate
IL-2Rα
2-Oxoisocaproate
2-Hydroxyisovalerate
IL-6
Glucose
MCP-1(MCAF)
LIF
SAA
Glutamine
HGF
Carnitine
Taurine
2-Oxoglutarate
M-CSF
Glycine
MIG
CTACK
Threonine
cis-Aconitate
TRAIL
Malonate
Eotaxin
Pyruvate
Mannose
Lysine
Alanine
Glycerol
Phenylalanine
Ornithine
IL-16
Haptoglobin
CRP
IFN-α2
IP-10
IL-3
SDF-1α
A2M
Creatinine
SAP
Creatine phosphate
Ferritin
IL-18
SCGF-β
SCF
PCT
tPA
